# Effects of sound exposure from a seismic airgun on heart rate, acceleration and depth use in free-swimming Atlantic cod and saithe

**DOI:** 10.1093/conphys/coz020

**Published:** 2019-05-16

**Authors:** Jan G Davidsen, Hefeng Dong, Markus Linné, Mathias H Andersson, Adam Piper, Tanya S Prystay, Eivind B Hvam, Eva B Thorstad, Frederick Whoriskey, Steven J Cooke, Aslak D Sjursen, Lars Rønning, Tim C Netland, Anthony D Hawkins

**Affiliations:** 1 NTNU University Museum, Norwegian University of Science and Technology, Trondheim, Norway; 2 Department of Electronic Systems, Norwegian University of Science and Technology, Trondheim, Norway; 3 FOI, Swedish Defence Research Agency, Stockholm, Sweden; 4 Institute of Zoology, Zoological Society of London, United Kingdom; 5 Fish Ecology and Conservation Physiology Laboratory, Department of Biology and Institute of Environmental and Interdisciplinary Sciences, Carleton University, Ottawa, Canada; 6 Thelma Biotel AS, Trondheim, Norway; 7 Norwegian Institute for Nature Research, Trondheim, Norway; 8 Ocean Tracking Network, Dalhousie University, Halifax, NS, Canada; 9 The Aquatic Noise Trust, Kincraig, Blairs, Aberdeen, United Kingdom

**Keywords:** Anthropogenic noise, fish behaviour, oil and gas exploration, particle motion, sound pressure, stress

## Abstract

Airguns used for offshore seismic exploration by the oil and gas industry contribute to globally increasing anthropogenic noise levels in the marine environment. There is concern that the omnidirectional, high intensity sound pulses created by airguns may alter fish physiology and behaviour. A controlled short-term field experiment was performed to investigate the effects of sound exposure from a seismic airgun on the physiology and behaviour of two socioeconomically and ecologically important marine fishes: the Atlantic cod (*Gadus morhua*) and saithe (*Pollachius virens*). Biologgers recording heart rate and body temperature and acoustic transmitters recording locomotory activity (i.e. acceleration) and depth were used to monitor free-swimming individuals during experimental sound exposures (18–60 dB above ambient). Fish were held in a large sea cage (50 m diameter; 25 m depth) and exposed to sound exposure trials over a 3-day period. Concurrently, the behaviour of untagged cod and saithe was monitored using video recording. The cod exhibited reduced heart rate (bradycardia) in response to the particle motion component of the sound from the airgun, indicative of an initial flight response. No behavioural startle response to the airgun was observed; both cod and saithe changed both swimming depth and horizontal position more frequently during sound production. The saithe became more dispersed in response to the elevated sound levels. The fish seemed to habituate both physiologically and behaviourally with repeated exposure. In conclusion, the sound exposures induced over the time frames used in this study appear unlikely to be associated with long-term alterations in physiology or behaviour. However, additional research is needed to fully understand the ecological consequences of airgun use in marine ecosystems.

## Teaser

A controlled short-term field experiment was performed to investigate the effects of sound exposure from a seismic airgun on the physiology and behaviour of two socioeconomically and ecologically important marine fishes. We determined that the sound exposures used in this study were unlikely to be associated with long-term alterations in physiology or behaviour.

## Introduction

Anthropogenic underwater sound generated by ships, power generation, oil and gas production, fishing, aquaculture and other industries is a growing concern as it may have negative impacts on fish and other marine organisms (Popper and Hastings, 2009; Boyed *et al.*, 2011; Normandeau Associates Inc., 2012; Hawkins *et al.*, 2015). Airguns used for offshore ocean seismic exploration by the oil and gas industry generate acute, repetitive, intense sounds (Hawkins *et al.*, 2014; Popper *et al.*, 2014). The airgun’s omnidirectional sound impulse has its greatest energy at low frequencies (20–50 Hz; Nieukirk *et al.*, 2004; Laws and Hedgeland, 2008). In seismic surveys, airguns are towed behind vessels and fired at the seabed at regular intervals (e.g. every 10–15 s). The reflected sound is detected by hydrophone arrays streamed behind the vessel (‘streamers’; Caldwell and Dragoset, 2000; Gisiner, 2016).

Depending on sound intensity and proximity, fish subjected to the noise from seismic airguns may incur physical damage (McCauley *et al.*, 2003), physiological stress responses (Sierra-Flores *et al.*, 2015) and/or exhibit behavioural changes (Løkkeborg and Soldal, 1993). The latter may include startle responses and flight (Wardle *et al.*, 2001) and reactions like moving to unaffected areas (Løkkeborg and Soldal, 1993). Airgun sounds have been associated with decreased fishery catch rates, perhaps caused by the displacement of fishes (Skalski *et al.*, 1992; Engås and Løkkeborg, 2002; Streever *et al.*, 2016). Physiological stress responses or behavioural reactions may inhibit activities like feeding and spawning and, hence, negatively impact fish populations. With globally increasing levels of underwater anthropogenic noise, managers need better information about the impacts of sound exposure on fishes (Slabbekoorn *et al.*, 2010; Hawkins *et al.*, 2015). In the case of repeated exposures, assessing cumulative effects is needed to determine relationships between dose and response and to design mitigation measures.

In this short-term field study, we used free-swimming Atlantic cod (*Gadus morhua*) and saithe (*Pollachius virens*) kept in a large sea cage (50 m diameter; 25 m depth). The fish were equipped with heart rate and body temperature biologgers and acoustic body acceleration and depth sensing transmitters. This permitted us to investigate the effects of sound from a seismic airgun on individual physiological responses and behaviour. The null hypothesis tested was that sound pressure and particle motion (PM) from the airgun would not alter heart rate, body acceleration, swimming depth or position of the fish in the cages.

## Materials and methods

### Fish capture and tagging

In total, 20 Atlantic cod {total length [mean ± standard deviation (SD)]: *T*_F_ = 556 ± 111 mm, range: 380–730 mm; mass: 1824 g ± 1000 g, range: 560–3820 g}, and 11 saithe (*T*_F_ = 431 ± 97 mm, range: 350–710 mm; mass: 571 ± 141 g, range 400–600 g) were captured near the study site with traps (*n* = 10 Atlantic cod) or rod and line (*n* = 10 Atlantic cod and 11 saithe) and equipped with electronic tags. All Atlantic cod were tagged with a heart rate (*f*_H_, beats per minute) and temperature data logger (Star-Oddi, Reykavik, Iceland; model DST Milli-HRT V10; 8 g in air; 13.0 × 39.5 mm). The logger was programmed to record body temperature and *f*_H_ at 100 Hz every 2 min and recordings of the electrical activity of the heart [electrocardiogram (ECG)] every 12 min. Eight of the Atlantic cods were also equipped with an acoustic accelerometer and depth-sensing transmitter to monitor fish movement (ThelmaBiotel, Trondheim, Norway; model AD-LP7; 2.1 g in air; 7.3 × 23.0 mm; power output: 137 dB re 1uPa @1m; duty time: 70–90 s; delay of transmission of sensor data: 4 h; acceleration sensor range/resolution: 0–3.465/0.01 ms^-2^; depth sensor range/resolution: 0–51/0.2 m). Only two saithe were large enough for the *f*_H_ loggers, and they were too small to be tagged with an additional transmitter. Nine saithe were tagged with acoustic transmitters only, using the same programming as for the cod. The surgical procedures were performed 8–11 days before the sound exposure.

The *f*_H_ was measured by implanting a leadless single-channel ECG-derived heart rate data logger with the electrodes embedded in the housing material. The logger recorded a burst measurement (600 samples) every second minute and stored the mean *f*_H_ with an accompanying quality index (QI). Every seventh measurement of the ECG was stored, enabling validation of the *f*_H_ and QI calculations.

Acoustic transmitters used on the cod and saithe contained accelerometer sensors that measured gravity forces and body acceleration in the *x*-, *y*- and *z*-planes, recording the tilt and roll angle of the fish, and motion in the forward and lateral directions. Acceleration was continuously measured for 60 s, after transmission of depth data.

After capture, the fish were held in a keep net (5 m length; 1 m width) for up to 12 h until tagging. The fish were anaesthetised (2-phenoxyethanol at 0.4 ml L^-1^; SIGMA Chemical Co., USA) and total body length (*T*_F_) was measured. Gills were irrigated with seawater during surgery (3–5 min). The *f*_H_ logger was inserted via a 5 cm longitudinal incision made halfway between the pectoral and pelvic fins, posterior to the pericardial membrane similar to the methods reported in Prystay *et al.* (2017). The *f*_H_ logger was inserted posterior to the pericardial membrane, with the upper part sutured to the ventral musculature. Incisions were closed using three independent sutures (non-absorbable monofilament; RESORBA Wundversorgung GmbH & Co. KG, Nürnberg, Germany; 5/0 Resolon). Acoustic tags were inserted into the body cavity on the ventral surface anterior to the pelvic girdle through a 1.5–2.0 cm incision (Davidsen *et al.,* 2013). The incision was closed using two independent sutures. Separate incisions were made for the *f*_H_ logger and acoustic transmitter in double-tagged individuals.

**Figure 1:**
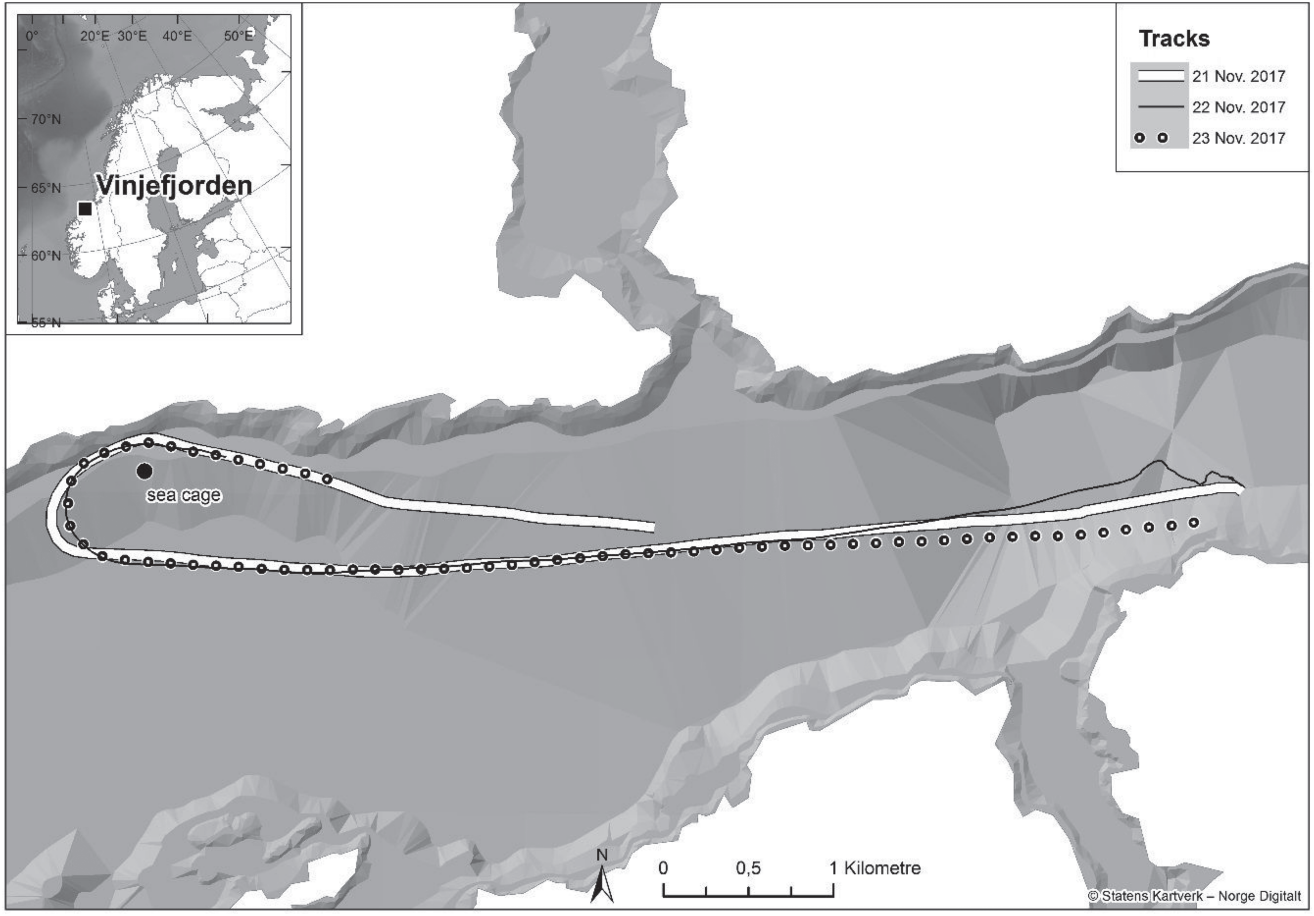
Location of the sea cage (large black dot) containing tagged Atlantic cod and saithe. The three tracks denote the path of the vessel towing the airgun during sound exposure experiments during 21–23 November 2017. The towing started at the easternmost end of the tracks.

After recovery, the fish were released into a sea cage (50 m diameter; 25 m depth) moored at the sea surface in Vinjefjorden, central Norway (depth of water column, 125 m; Fig. 1). The procedures followed national ethical requirements and were approved by the Norwegian National Animal Research Authority (permit number 13647).

### Recording of data from fish tags

Eight receivers (Thelma Biotel, AS, Trondheim, Norway, Model TBR 700) recording signals from the acoustic transmitters were deployed 2.5 and 4.5 m below the surface at the outer ring of the sea cage (supporting information Fig. S1). To minimize signal collision from the transmitters, two frequencies (10 tags on 69 kHz and 7 tags on 71 kHz) were used. One synchronization tag (Thelma Biotel; model ART MP-13) for each frequency was deployed to correct for drift in receiver clocks.

### Sound exposure

The seismic airgun sound exposures included both sound pressure and PM components. A commercial airgun (Bolt Longlife Southampton, PA, USA; model 1900 LLXT; 40 cubic inches; www.bolt-technology.com) was rigged on board the vessel MS Harry Borthen and operated by the Norwegian Geological Institute (www.ngi.no). The airgun was towed 11 m behind the boat at 4 m depth. The sound exposures were performed during 21–23 November 2017 (supporting information Table S1). Each day started with a simulation of ramp up (a gradual build-up of airgun sound level over time, usually 20–40 min), achieved by moving the boat closer to the sea cage. The towing of the airgun started 6.7 km from the sea cage, with one shot every 10 s (vessel speed, 8.3 km h^-1^; Fig. 1). The closest distance between the airgun and the fish was 100 m.

Each day, immediately after the end of the towed shooting, the boat turned back towards the experimental cage with the airgun towed at same speed and depth, but with no further shooting, ending at a mooring location 200 m away from the sea cage. After a break (104, 168 and 87 min during the three days; duration of the breaks was dictated by weather conditions which varied among the days), the airgun was reenergized and shot every 10 s for a period of 10 min from this position (stationary shooting), with the airgun hanging straight down from the boat at 4 m depth. Due to currents, the ship with the airgun drifted around the mooring, so the vertical position during the shooting varied by 2–5 m. During the first two days, the main engine of the boat was turned off; however, the smaller engine generating on-board electricity was operating. During the third day, both engines were turned off. The pressure of the airgun differed among days due to low pressure in the nitrogen batteries (supporting information Table S1). The scientific use of the airgun was approved by the Norwegian Petroleum Directorate (permit number 672/2017).

### Measurement of *in situ* sound pressure and Particle motion

#### Sound pressure levels

The sound pressure levels from ambient noise and the airgun were measured at the test site. Three hydrophones, HTI-96-MIN (sensitivity, −170 dB re 1V/μPa) with the acquisition system SYLENCE Acoustic Recorder from RTSYS were deployed; two on the eastern side of the sea cage at 18 m and 20 m depth and a third on western side at 18 m depth (supporting information Fig. S1). On day two, the recorder on the western site failed. The ambient sound level before, during and after the experiment was recorded to establish a baseline for what the fish were exposed to in the absence of seismic shooting. These recordings were also used to determine the signal-to-noise ratio for the seismic shooting. Second, the sound pressure at the sea cage was recorded when the airgun was operating. The hydrophones attached to the sea cage were saturated when the airgun was closer than 2 km, so data measured by the built-in hydrophone in the vector sensor (see below) were used for these analyses.

#### Particle motion

The particle acceleration levels were measured in terms of their three directional components (*x* and *y* axes pointing in the horizontal plane and *z* axis in the vertical plane). The PM sensor was lowered to 5 m depth from the sea cage ring (supporting information Fig. S1) and operated from 20 November at 18.00 h until 23 November 14.00 h UTC, except from 21 November 15.00 h to 22 November 05.00 h UTC because of inspection of sensor and data backup.

The particle motion sensor was a custom-built system based on the description in Sigray & Andersson (2011), but the sensor used was autonomous and the sphere was smaller (diameter of 0.06 m) and the sphere was kept suspended 0.3 m above the sensor platform. A PCB Piezotronics, model 356B18, 3-axis accelerometer was mounted inside the sphere, with a flat sensitivity in the frequency range (+/-5%) of 0.5 Hz–5.0 kHz. The sensitivity of the accelerometer was 1 V/g, g being the gravitational constant of ~9.82 m/s^2^. The sensor’s noise floor at 10 Hz was 4 μg/Hz1/2 = 32 dB re 1 μm/s^2^, and at 100 Hz 1.2 μg/Hz1/2 = 22 dB re 1 μm/s^2^. The sampling frequency was 14 400 Hz, and the resolution of the Analog-to-Digital converter was 24 bit. The sensor had a sound pressure hydrophone (Cetacean C55RS, sensitivity −180 dB re 1V/μPa) connected to the data acquisition system.

### Environmental variables

During sound measurement, conductivity and temperature at different depths (CTD) were measured using an SD204 CTD-sonde (Saiv AS, Bergen, Norway).

### Video recording of fish

To enable visual observation of fish behaviour during the experiment, two underwater video cameras with built-in infra-red LEDs (Sony CCD, Model 37CSHR-IR) were deployed in a small net pen (3 m diameter; 4 m depth) within the larger sea cage (supporting information Fig. S1). One was an up-looking camera fixed in the centre of the small net-pen floor. The other was a down-looking camera fixed at the surface. Both cameras were connected to a Digital Video Recorder (ABUS 4-channel TVVR30004). An accompanying down-looking camera (GoPro Hero4) was fixed at the surface to supplement the daytime footage. Eleven additional untagged saithe (*T*_F_ < 400 mm) captured using the same methods as tagged fish within the main experiment were released into the video net pen on 20 November. Cameras were set to record continuously for the entire study period. Footage was collected at 25 frames per second from 19.00 h on 20 November to 14.00 h on 22 November. Due to bad weather causing a power loss, no footage was collected on 23 November.

### Data analyses

#### Sound pressure and particle acceleration motion

To relate the biological data to the sound data (supporting information Table S1), a series of acoustic metrics were calculated, each commonly used to describe an airgun pulse. The ISO 18405:2017 (International Organization for Standardization (2017)) standard was used as a foundation for the calculations (supporting information Table S2). Our acceleration exposure density level and the zero to peak acceleration level metrics do not have clear ISO definitions and were calculated similarly to how pressure is calculated by ISO (18405 chapters 3.2.1.9 and 3.2.2.1; supporting information Table S2).

In the ISO standard, the acceleration exposure level (AEL) is defined as the time integrated squared sound particle acceleration. Here, the metric was calculated as the area under the curve of the acceleration exposure density level curve within the specified frequency range.

For each exposure, a 1 s-long time series was analysed. The time series was selected such that the peak pulse was in the beginning of the file, within 200 ms from the start (supporting information Fig. S2). Occasionally, a measurement had to be selected differently or discarded due to sharp transients from the accelerometer colliding with the data acquisition unit due to waves. The time series was bandpass filtered (third-order Butterworth filter) in the frequency range of 5–1000 Hz to minimize low frequency waves, and the upper limit was the maximum frequency with reliable data from the PM sensor.

Using the 1 s time series segment, the zero-to-peak value emanating from the airgun pulse was estimated by finding the maxima or minima of the signal relative to the baseline (zero) and taking its absolute value. Thereafter, the sound pressure and particle acceleration exposure spectral density (ESD) was calculated with 1 Hz bin width. Integrating the sound ESD bins from 5 to 500 Hz gives the sound exposure level (SEL_sp_; sp means single pulse). By summing up the particle acceleration ESD frequency binwise for the three accelerometer outputs and thereafter integrating the data over the frequency range gives AEL_sp_ for the total acceleration field. The SEL and AEL correspond to the sensor-measured sound pressure and particle acceleration energy. By summing up the energies from each pulse, the cumulative (_cum_) sound pressure and particle AELs were achieved (SEL_cum_ and AEL_cum_, respectively).

The background measurements were calculated as described above. A 1 s-long time series was measured 1–2 s prior to exposures. Comparing background zero-to-peak data with the exposure test must account for the possibility that the background data may contain short transients/bursts from surface waves. Comparing SEL and AEL is appropriate since the transients are averaged out in the analysis.

#### Quality of data from fish tags

Measurement of heart rate was successful with >89% of the data classified as Q0/Q1 (very good/good) and 11% of the data discarded (analyses using the Mercury software, V4.64; Star-Oddi, Reykavik, Iceland). One *f*_H_ logger had technical problems. The acceleration sensor failed in two acoustic tags, so body acceleration data were obtained from seven cod and seven saithe, while data on swimming depth and location within the sea cage were collected for eight fish from each species. Data from the pressure sensor were corrected for daily changes in atmospheric pressure.

#### Horizontal position of fish within the sea cage

Positions of fish carrying acoustic tags in the sea cage were calculated using multilateration based on time difference of arrival of tag signals to the receivers, corrected for drift of the receiver clocks and variation in temperature (software tool PinPoint; Thelma Biotel).

#### Effect of sound exposures on fish behaviour

Change in heart rate, measured as deviations from resting level (average of the lowest 10% of the heart rate values recorded during 19–24 November 2017; see supporting information Table S3 for resting values for individual fish), was determined. Change in depth, body acceleration and position in the sea cage (recorded as distance from origo, i.e., centre of the cage defined as a vertical vector that extended from the surface to the bottom of the cage) from resting states was determined. The change in these values over specified time periods were used as independent variables in separate linear mixed effects models with individual fish as a random effect, to determine whether heart rate, body acceleration, depth or distance to origo varied in relation to the measured sound metrics (supporting information Table S2) or over the three days, and to test if any types of sound exposure (towing no shooting, towing and shooting and stationary shooting) had an effect on heart rate and behaviour. Tukey post-hoc tests were used to identify differences between days. Fish body temperature was compared between species and day using a linear mixed effect model, with individual fish as a random effect to account for repeated measures (R-nlme package; Pinheiro *et al.*, 2014).

Random forest analyses were used to determine the top three best explanatory response variables (four models per species; heart rate, body acceleration, depth and distance to origo). For cod, two random forest analyses were performed, one including all the data (i.e., heart rate, body temperature, depth, body acceleration, distance from origo, sound metrics and type of sound exposure) and the second including sound and body temperature, to assess the effects of sound only. For saithe, only the second random forest analysis was used since each fish was tagged with one tag. Linear mixed effect models with individual fish as a random effect and including the top three variables that explained most of the variation were used to assess the significance of each relationship. Stepwise model selection using Akaike information criterion (AIC) was used to further simplify the models.

**Figure 2:**
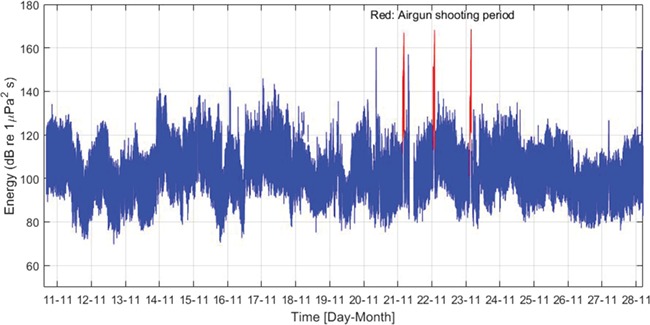
Energy level (blue: background noise; red: airgun shots) calculated by integrating the spectrogram values from 5 to 500 Hz as a function of time in Vinjefjorden during 11–28 November 2017.

**Figure 3:**
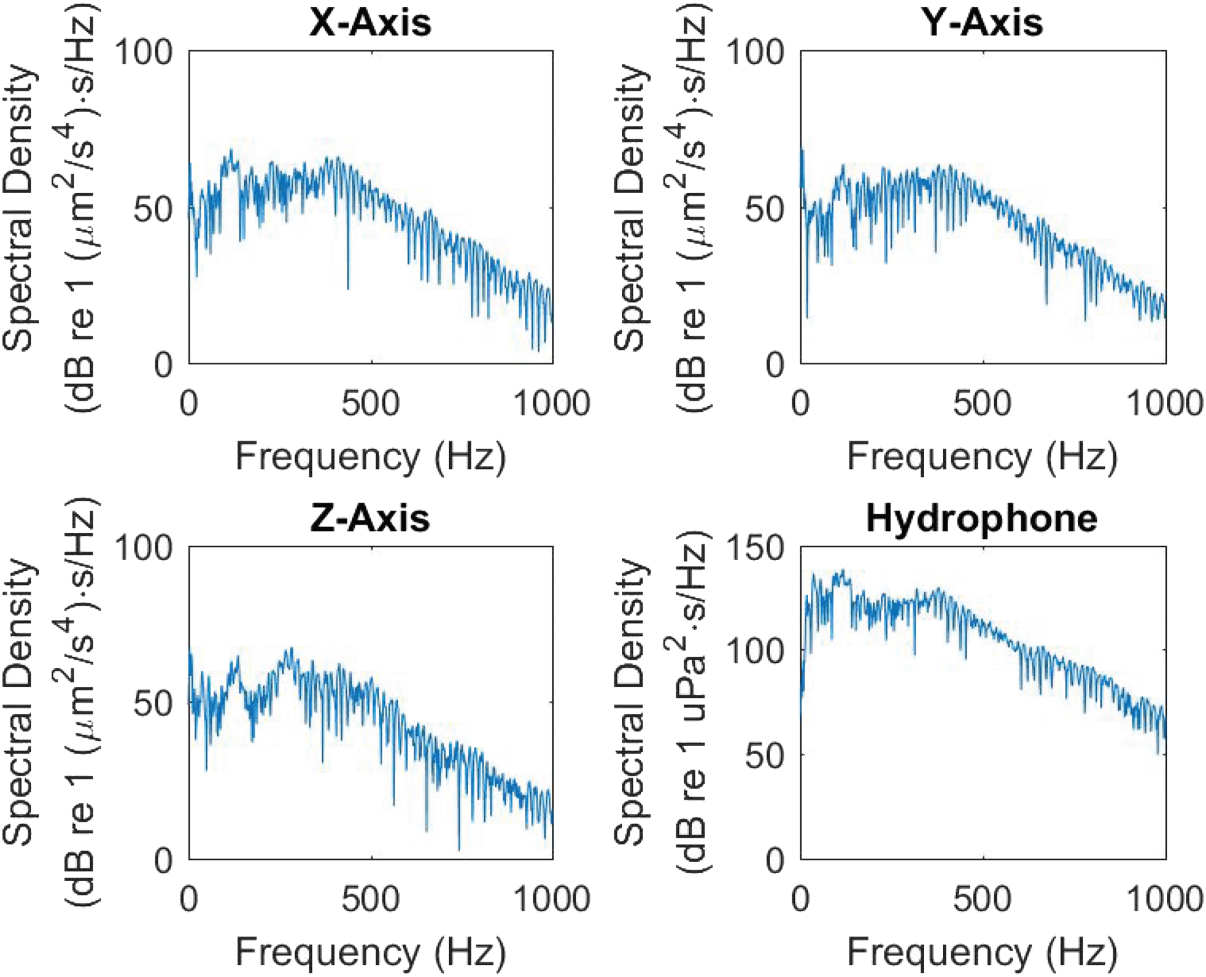
The sound pressure and particle acceleration ESDs at the sea cage for an airgun pulse from an airgun 200 m away during the stationary shooting on day 1.

Since body temperature impacted all variables, the effect of temperature on the response variables was detrended by determining the residuals of the relationship between the variable (i.e., heart rate, body acceleration, depth or distance to origo; Jakob *et al.*, 1996). The residuals served as the response variable in the same linear mixed effect models using the covariates that were significant in the previous tests and their interaction with time to determine how fish responses changed over the study period. Residuals were only used in post-hoc analyses (Freckleton, 2002).

### Video footage of fish behaviour

Footage from each sound exposure and 1 h preceding each exposure was analyzed, and for each visible fish the following were quantified: (i) body alignment relative to the water flow direction (degrees) derived from particle movement in the water, with values from 0 (perfectly aligned) to 180^o^ (totally opposite); (ii) nearest neighbour distance (a measure of shoaling cohesiveness), measured from the centre of the head; and (iii) approximate swimming depth (near surface, midwater or near cage base). Metrics were recorded every 10 min during pre-exposure and towing and shooting. For towing without shooting and stationary shooting, metrics were recorded at the start, middle and end of each period. Generalized linear models with a quasi-poisson error distribution were used to test for exposure and day effects on fish alignment to flow and nearest neighbour distance. A Chi-squared test was used for the swimming depth data, with each day analysed separately. Sampled footage was also searched for changes in fish behaviour indicative of a startle or stress response such as a rapid acceleration in swimming speed or abrupt change in direction.

## Results

### Environmental variables

During the first two days, the surface water temperature was 8.0°C, increasing with depth to 11.5°C at 15 m depth. Salinities were 30‰ at the surface and 32‰ at 15 m depth. A storm in the night between the second and third day lowered temperatures to 7.3°C at the surface and 10.0°C at 15 m. Salinity did not change.

**Table 1:** Overview of the sound pressure and particle acceleration during the different sound exposures including background levels, measured at the sea cage (start means the first shot)

		P_0-pk_ (dB re 1 μPa)		SEL_sp_ (dB re 1 μPa^2^·s)		SEL_cum_ (dB re 1 μPa^2^·s)		A_0-pk_ (db re 1 μm/s^2^)		AEL_sp_ (dB re 1 (μm/s^2^)^2^·s)		AEL_cum_ (dB re 1 (μm/s^2^)^2^·s	
Type of exposure	Exposure number	Start	CPA	Start	CPA	Start	CPA	Start	CPA	Start	CPA	Start	CPA
Transect and shooting	1	152	181	133	157	142	173	97	115	83	92	88	111
Transect background	1	119	127	107	116	115	134	87	90	77	79	83	99
Transect and shooting	4	158	183	134	158	139	172	106	121	84	108	89	125
Transect background	4	133	137	120	120	121	150	105	122	94	110	95	125
Transect and shooting	7	150	185	129	158	129	173	91	121	71	94	71	111
Transect background	7	119	125	105	111	105	134	84	91	67	78	67	95
Stationary and shooting	3	176	NA	152	NA	159	172*	116	NA	91	NA	96	109*
Stationary background	3	123	NA	110	NA	110	127*	98	NA	83	NA	77	93*
Stationary and shooting	6	180	NA	157	NA	166	174*	116	NA	95	NA	93	113*
Stationary background	6	149	NA	132	NA	126	138*	124	NA	111	NA	103	114*
Stationary and shooting	9	181	NA	158	NA	166	175*	119	NA	92	NA	100	109*
Stationary background	9	124	NA	108	NA	116	125*	82	NA	70	NA	71	83*

^*^This is the cumulative value for the complete shooting session.

**Figure 4:**
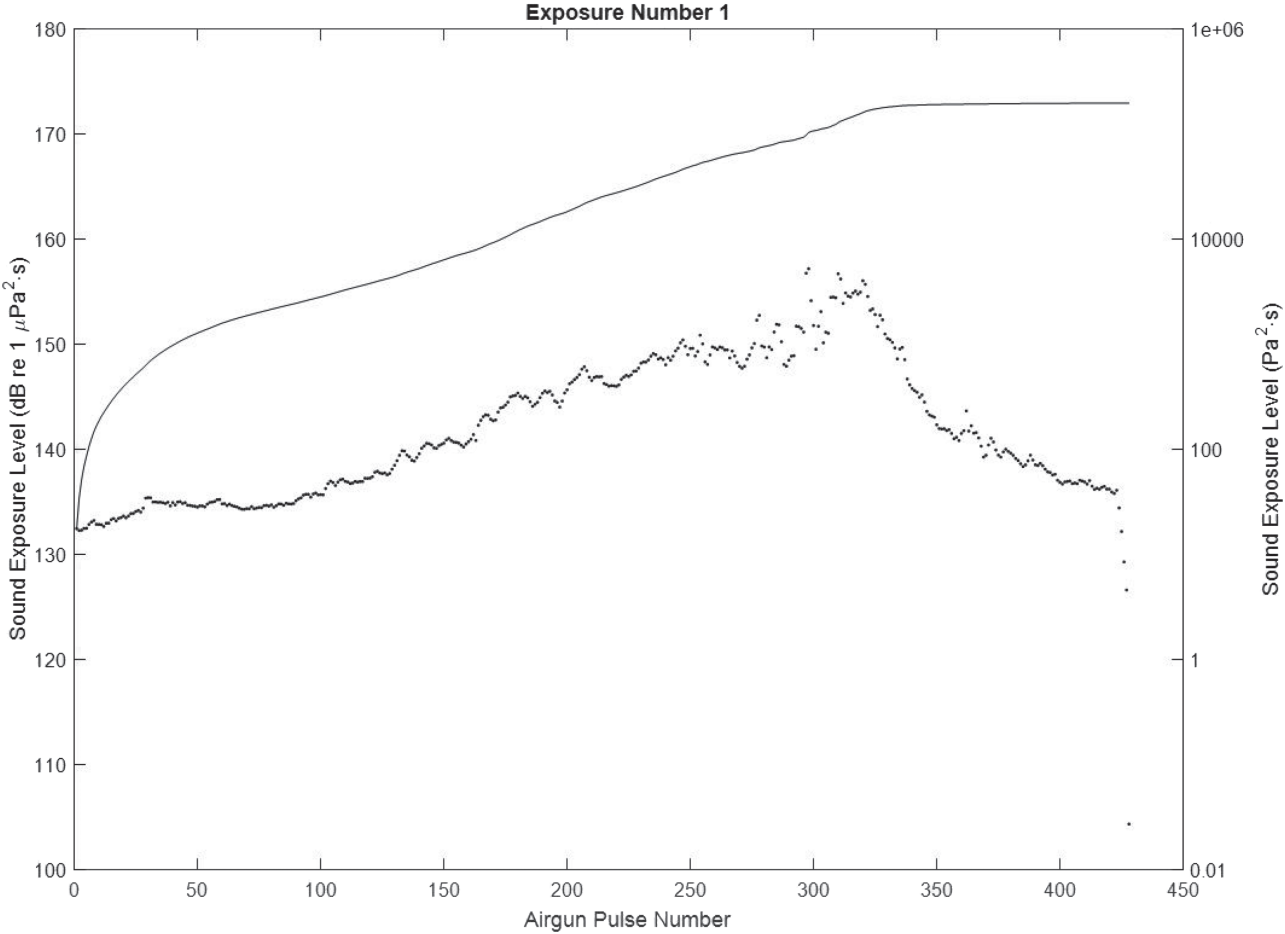
SEL (single pulse and cumulative) during exposure 1 (day 1), when the airgun was moving towards the sea cage. The dots represent the SEL_sp_ and the curve the SEL_cum_.

**Figure 5:**
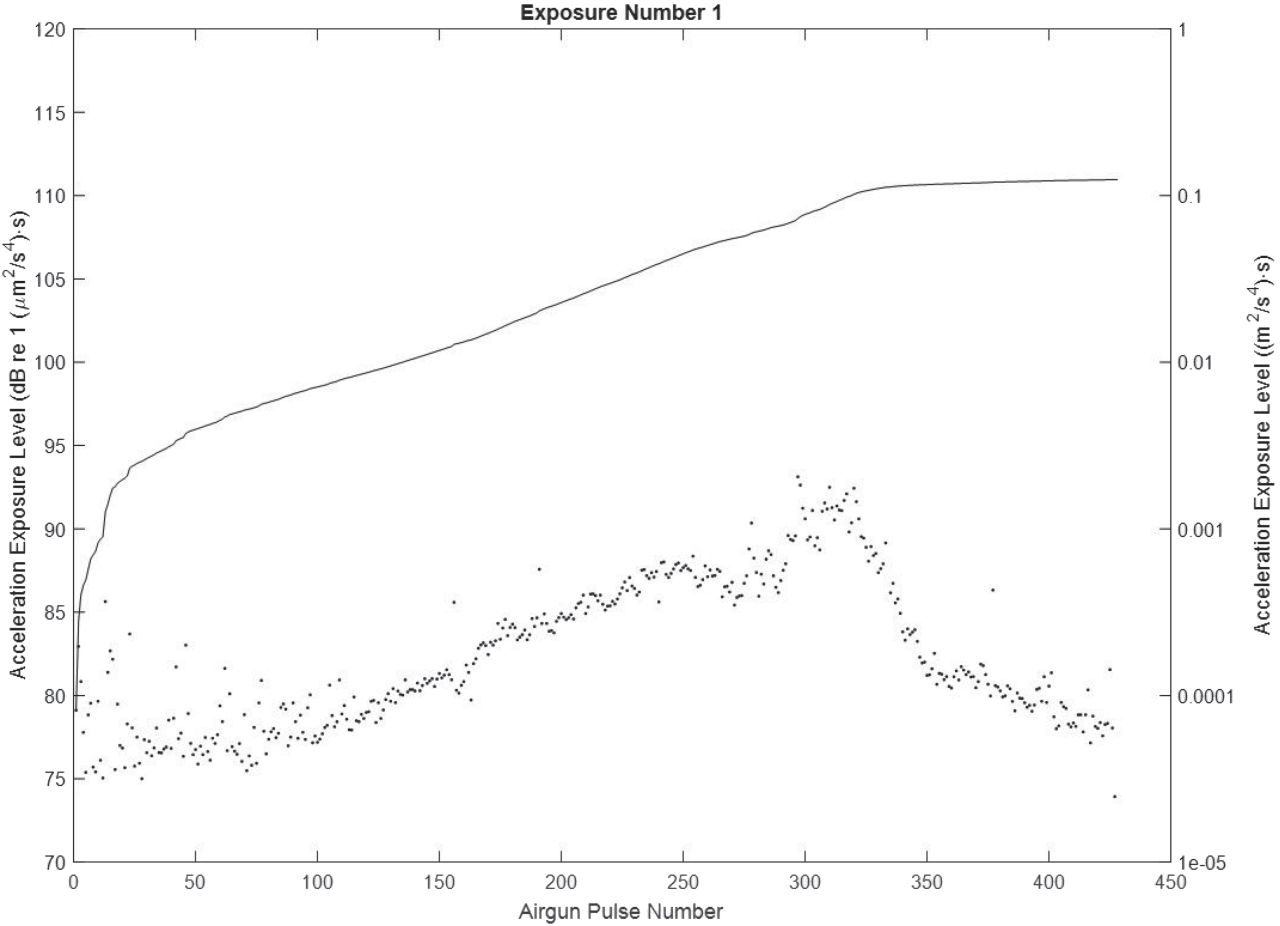
Particle AEL (single pulse and cumulative) during exposure 1 (day 1), when the airgun was moving towards the sea cage. The dots represent the AEL_sp_ and the curve the AEL_cum_.

### Sound exposure

Ambient noise levels in terms of rms (root mean square) pressure in 1 s intervals were high and variable. The mean of the rms sound pressure level in the whole period without airgun shooting was 103 dB re 1 μPa (SD ± 11 dB re 1 μPa). The energy level (Fig. 2) varied over time in a similar pattern as the sound pressure level.

The PM sensor registered all sound exposures, both sound pressure and PM, during the three experimental days. The sound pressure and particle acceleration at the sea cage showed an initial sound pulse, followed by reflections (supporting information Figs S3 and S4). The *x*-axis showed the highest acceleration. Sound and acceleration ESDs showed that the received noise had most energy below 500 Hz for both sound pressure and PM (Fig. 3).

**Figure 6:**
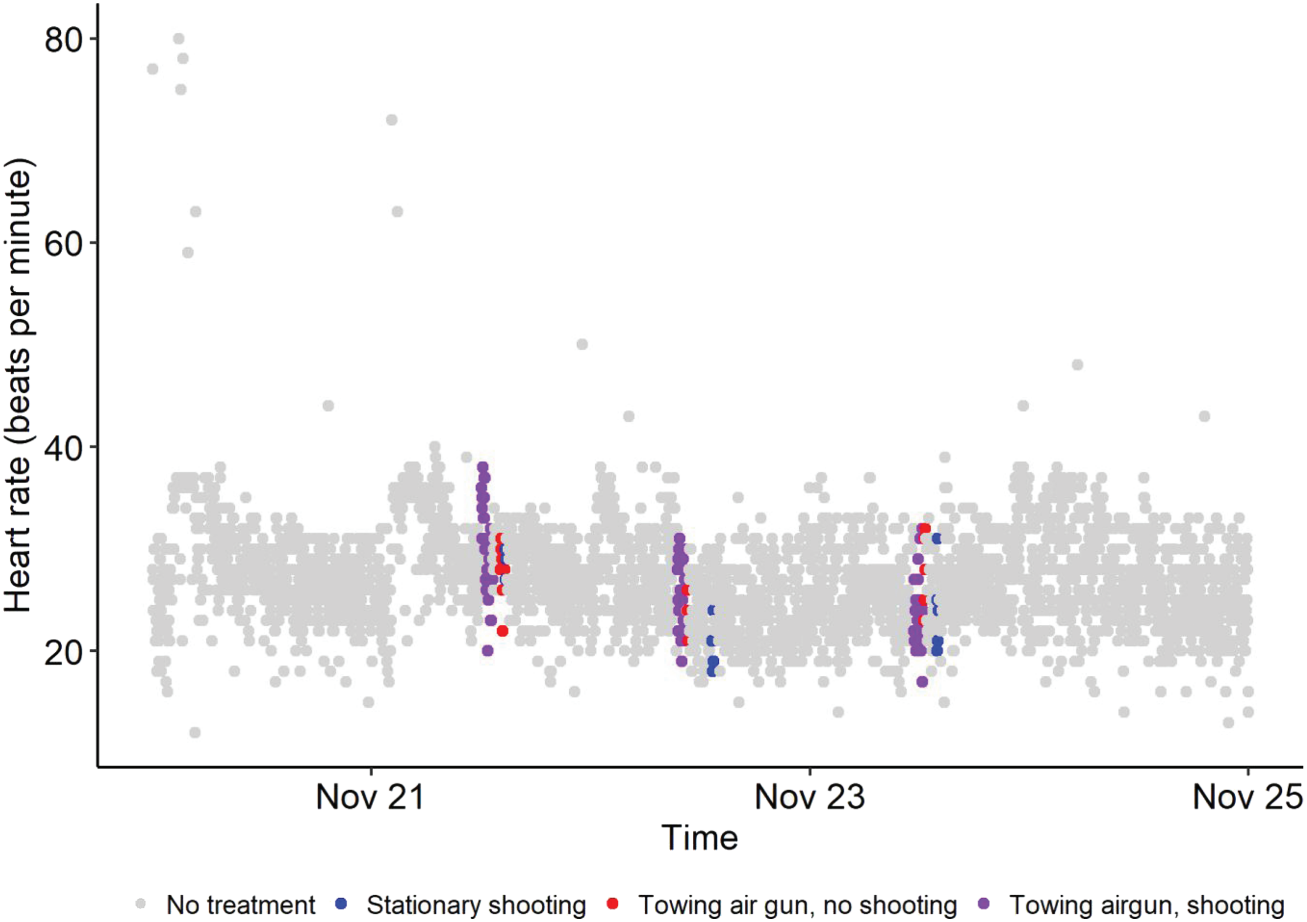
Example of heart rate trace recorded for one individual starting from midnight 20 November 2017 and ending midnight 25 November 2017 (see supporting information Fig. S13 for heart rate traces from each individual).

The sound level (SEL and AEL) when the airgun fired its first shot during the towed transect was detectable at the sea cage (Table 1, Figs 4 and 5 and supporting information Figs S5, S6, S9 and S10). As the vessel came closer, the sound level increased up to a maximum at the closest point of approach (CPA; 100 m, estimated from P_0-pk data_) and decreased when the vessel moved away (Figs 5 and 6). The received P_0-pk_ and SEL (single pulse and cumulative) for this transect were well above the background levels and is representative for the other exposures (Fig. 3, Table 1 and supporting information Figs S5, S6, S9 and S10). The difference between the pulse and background levels was between 18 and 60 dB, depending on the metric. However, for A_0-pk_ and AEL (single and cumulative), exposure events number 4 and 6 on day 2 showed similar levels for the airgun’s pulse and background sound (Table 1). This is due to rough weather conditions on day 2, which agitated the accelerometers with water motion. The time when the P_0-pk_ values peaked was used for checking that the A_0-pk_ pressure measurements were less sensitive to surface effects.

When the vessel was stationary 200 m away from the sea cage, the sound level did not vary over time until the airgun’s air pressure was reduced (supporting information Figs S3, S4, S7, S8, S11 and S12). Since the stationary exposure did not have a CPA, the CPA values in Table 1 show the cumulative value for the shooting session. Similar to the towed shooting, the airgun pulses were well above background levels for sound pressure throughout the stationary exposures. The difference between the pulse and background levels was between 25 and 57 dB, dependent on the metric. Exposure 6 showed the same weather-dependent levels for A_0-pk_ and AEL (both single and cumulative) as exposure 4 (supporting information Figs S5–S8).

### Heart rate

**Figure 7:**
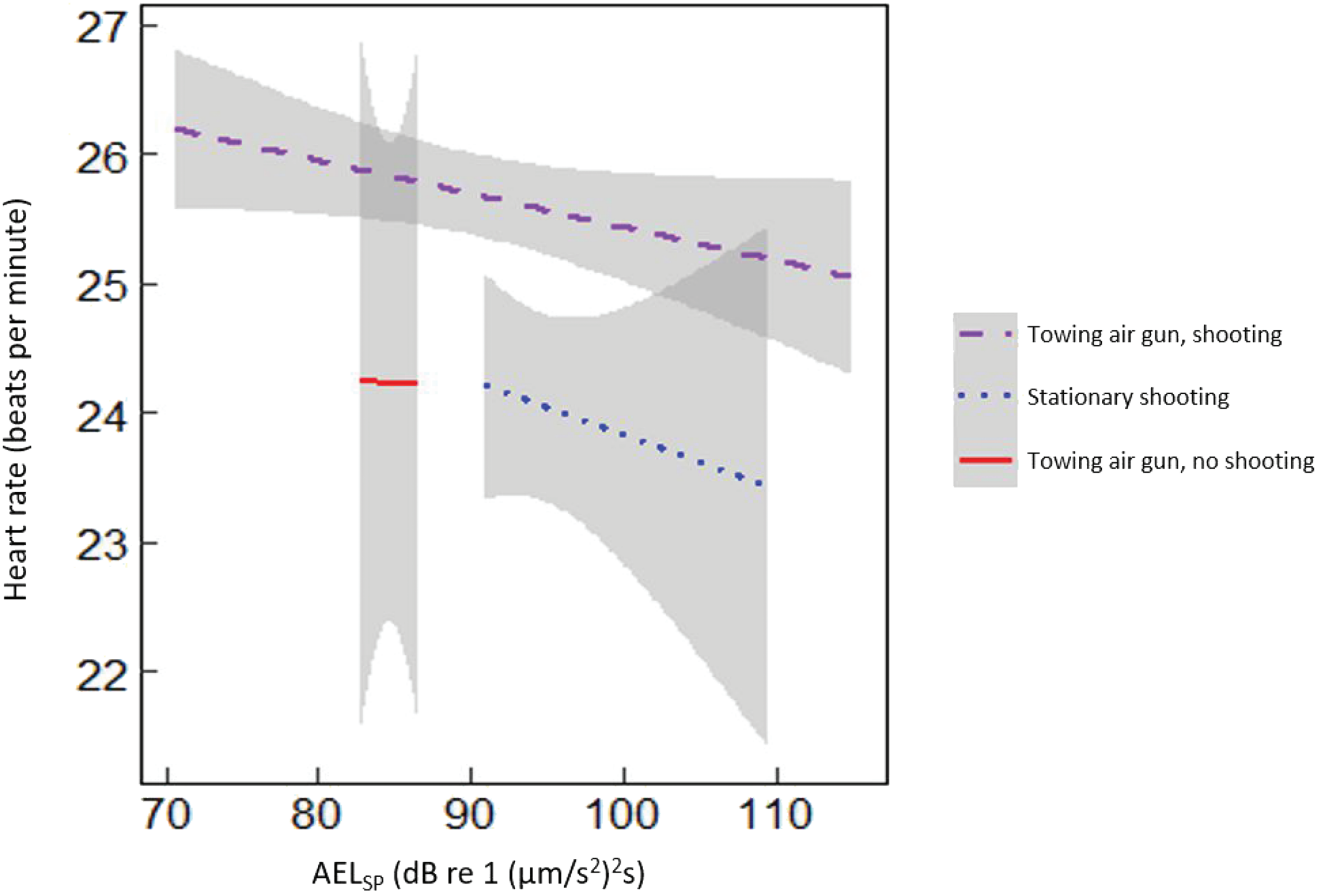
Changes in cod (*n* = 20) heart rate during three types of sound exposure. The grey areas indicate the 95% confidence interval of the linear model.

An example heart rate trace is presented in Fig. 6 (see supporting information Fig. S13 for heart rate traces from each individual). Bradycardia was observed during sound exposure for most cod but was not long lived, and there was little evidence of tachycardia or arrhythmia during the exposure or recovery period. In the random forest model containing all covariates, the three most important factors affecting cod heart rate were swimming depth, distance to origo and body temperature (Table 2). The percentage of variance explained was only 8.6%, suggesting low predictive power for this model. Changes in heart rate from resting were positively related to swimming depth. When behavioural factors (depth, body acceleration and distance to origo) were excluded, the top three factors influencing cod heart rate (all with negative relationships) were body temperature, AEL_sp_ and AEL_cum_. When broken down by the type of sound exposure, the trend remained negative both for shooting while towed and stationary shooting but not for towing without shooting (Fig. 7). In the two saithe tagged with heart rate data loggers, changes in heart rate were positively related to body temperature (Table 3).

### Swimming depth

The change in cod swimming depth was positively related to heart rate and negatively related to body temperature and distance to origo (Table 2). When behavioural data were excluded, the top three factors associated with changes in swimming depth were body temperature and SEL_sp_ and SEL_cum_. However, only the negative relationship with body temperature was significant.

In saithe (Table 3), the top three factors that most (63% of variance explained) influenced the change in depth were AEL_sp_ and AEL_cum_ and SEL_sp_. Due to a lack of measurements, heart rate and body temperature were not included. Swimming depths increased during both towed and stationary shootings (Linear mixed effects models (lme); *n* = 8; *P* < 0.001) but not when the airgun was towed without shooting (*n* = 8; *P* > 0.05).

### Body acceleration

In cod, the random forest analysis identified the type of sound exposure (shooting while towed, stationary shooting and towing without shooting), SEL_sp_ and A_0-pk_ as the top three factors that influenced the change in body acceleration (10% of variance explained); however, none of the covariates tested were significant predictors of change in body acceleration (Table 2).

In saithe (Table 3), random forest analysis identified three sound variables (P_0-pk_, SEL_sp_ and AEL_cum_) that accounted for 19% of the variance in change in body acceleration. The linear mixed effect model showed a positive significant relationship between body acceleration and P_0-pk_ and a negative relationship with SEL_sp_. Due to a lack of measurements, heart rate and body temperature were not included.

### Position in sea cage

In cod, swimming depth, SEL_cum_ and heart rate were identified by random forest analysis as the top three factors accounting for most of the explained variance (46%) in change in distance to origo (centre of the sea cage). Swimming depth and and heart rate were negatively associated with distance from origo, while there was no significant relationship with the cumulative pulse SEL (Table 2). Excluding behaviour, the top three measurements that explained most of the variance in the change in distance to origo from resting were body temperature, P_0-pk_ and SEL_cum_ (25%), but only SEL_cum_ and body temperature had a significant relationship. Cod tended to move away from the origo as SEL_cum_ increased. Body temperature was negatively associated with distance from origo.

In saithe, the top three factors that explained change in distance to origo were AEL_sp_, SEL_sp_ and P_0-pk_ (57% of variance explained). However, distance to origo was only significantly related to AEL_sp_ (Table 3), with distance increasing with AEL_sp_. Due to lack of measurements, heart rate and body temperature were not included.

### Temperature detrended data

Due to the influence of body temperature, the same linear mixed effect models as above (Tables 2 and 3) were run using detrended temperature response variables and with the covariates related to day number of the sound exposure to determine in more detail how the responses differed over time (Table 4).

**Figure 8:**
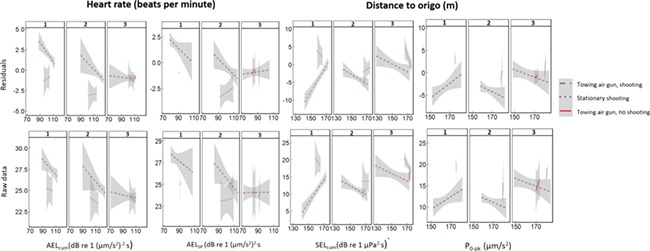
Significant (*P* < 0.05) temperature detrended (top row) and raw (bottom row) responses of cod (*n* = 8) to sound exposures (*x*-axis) identified using random forest analysis and linear mixed effect models. Significant variables were tested against temperature detrended data to determine whether the trend was driven by sound or was an artefact of temperature. Numbers 1–3 indicate the day number of sound exposure.

For cod, there were significant positive relationships with temperature-corrected heart rate and the interaction between AEL_cum_ and day (Table 4), day number of exposure and the interaction between distance from origo and day. There was no relationship with AEL_sp_; however, the interaction term with day number of exposure was significant (*P* < 0.001). Specifically, differences in heart rate decreased as days exposed to sound from the seismic airgun progressed (e.g., habituation; Fig. 8). The negative interaction between heart rate and sound exposures were significant for all sound metrics (Table 5).

There was no relationship between temperature-corrected cod swimming depth and sound exposure (all *P* > 0.05; Table 4), but there was a negative relationship between distance from origo and P_0-pk_ and SEL_cum_. For both cases, distance to origo increased during the shooting while towing and during stationary shooting on day 1 but decreased on days 2 and 3 (Fig. 8).

For saithe, there was no significant relationship between temperature-corrected heart rate and the sound exposures (all *P* > 0.05).

### Video observations of fish behaviour

Four to eight saithe were visible within the footage at each measurement. Alignment with the flow direction ranged from 0.7 to 176° (interquartile range: 3.9 – 15.0°). Fish were less aligned with flow during stationary shooting (*P* < 0.001; 50% of residual deviance), and there was also a day effect with the largest deviation from flow direction recorded on day 2 of the sound exposures (*P* < 0.001). Nearest neighbour distances ranged from 24 to 360 mm (interquartile range: 73.5 – 138.1 mm). Fish were more dispersed with significantly larger nearest neighbour distances during the sound exposures both during towing (*P* < 0.001; 5%) and stationary shooting (*P* < 0.001; 12.4%) than during towing only and the pre-exposure period. Swimming depth did not vary between exposures either during day 1 (Χ^2^ = 4.6; *P* = 0.59) or day 2 (Χ^2^ = 5.7; *P* = 0.46). Abrupt movements and changes in swimming direction were rarely observed, and there was no evidence of a C-start response.

**Table 2:** Linear mixed effect model results for cod (*n* = 8) heart rate, swimming depth, body acceleration and distance from the centre of the sea cage (origo). Correlations are given between sound measurements and heart rate, body acceleration and depth. Separate models were tested for each sound measurement

Response variable	Model type	Delta AICc_null_	Random forest percent variance explained (%)	Covariate name	Coefficient	SE	*P*
1) Heart rate	All covariates	2412	8.6	Swimming depth	0.270	0.110	**0.012**
				Distance to origo	−0.002	0.040	0.96
				Body temperature	0.015	0.200	0.94
	Behavioural factors excluded	9945	2.8	Body temperature	−10.700	0.970	**<0.001**
				AEL_sp_	−1.080	0.100	**<0.001**
				AEL_cum_	−0.027	0.001	**0.048**
2) Swimming depth	All covariates	1669	51.9	Body temperature	−0.560	0.085	**<0.001**
				Distance to origo	−0.270	0.014	**<0.001**
				Heart rate	0.069	0.020	**<0.001**
	Behavioural factors excluded	1702	33.1	Body temperature	−1.300	0.130	**<0.001**
				SEL_cum_	0.002	0.021	0.93
				SEL_sp_	−0.018	0.022	0.43
3) Body acceleration	All covariates		No good models and all covariates were *P* > 0.05				
	Behavioural factors excluded		No good models and all covariates were *P* > 0.05				
4) Distance from origo	All covariates	1269	45.7	Swimming depth	−1.300	0.110	**<0.01**
				SEL_cum_	0.033	0.026	0.21
				Heart rate	−0.190	0.068	**<0.01**
	Behavioural factors excluded	1827	25.1	Body temperature	2.000	0.360	**<0.001**
				Zero to peak sound pressure level (P_0-pk_)	−0.060	0.052	0.26
				SEL_cum_	0.100	0.048	**0.031**

**Table 3:** Linear mixed effect model results for saithe (*n* = 9) swimming depth, body acceleration and distance from the centre of the sea cage (origo). Correlations are given between sound measurements and heart rate, body acceleration and depth. Separate models were tested for each sound measurement

Response variable	Model type (*n* = no. fish)	Residual variance explained (%)	Covariate name	Coefficient	SE	*P*
1) Heart rate	Behavioural factors excluded (*n* = 2)	−25.7	Body temperature	3.700	0.380	**<0.001**
			Zero to peak acceleration level (A_0-pk_)	0.043	0.056	0.13
			AEL_sp_	−0.040	0.056	0.44
2) Swimming depth	Behavioural factors excluded (*n* = 8)	63.3	AEL_cum_	0.086	0.015	**<0.001**
			AEL_sp_	0.052	0.014	**<0.001**
			SEL_sp_	−0.077	0.011	**<0.001**
3) Body acceleration	Behavioural factors excluded (*n* = 7)	−18.7	Zero to peak sound pressure level (P_0-pk_)	0.015	0.007	**0.043**
			SEL_sp_	−0.017	0.007	**0.019**
			AEL_cum_	−0.001	0.002	0.52
4) Distance from origo	Behavioural factors excluded (*n* = 8)	56.6	AEL_sp_	−0.088	0.029	**<0.01**
			SEL_sp_	0.029	0.150	0.84
			Zero to peak sound pressure level (P_0-pk_)	0.110	0.140	0.45

**Table 4:** Linear mixed effect model results for temperature detrended cod (*n* = 8) and saithe (*n* = 2) heart rate, swimming depth, body acceleration and distance from the centre of the sea cage (origo). Correlations are given between sound measurements and the measured covariates for cod only due to sample size limitations. Separate models were tested for each sound measurement

Response variable (residuals)	Model type	Delta AICc_null_	Covariate name	Coefficient	SE	*P*
COD						
1) Heart rate	All covariates	2408	Swimming depth	0.230	0.100	**0.022**
			Distance to origo	−0.008	0.042	0.85
			Day	−1.600	0.920	0.094
			Swimming depth:day	−0.031	0.074	0.67
			Distance to origo:day	0.070	0.033	**0.035**
	Behavioural factors excluded	1926	AEL_sp_	−0.034	0.034	0.32
			AEL_cum_	0.043	0.028	0.13
			Day	1.900	0.570	**0.001**
			AEL_sp_:day	0.005	0.015	0.76
			AEL_cum_:day	−0.034	0.013	**<0.01**
2) Swimming depth	All covariates	1710	Distance to origo	−0.270	0.014	**<0.001**
			Heart rate	0.065	0.020	**<0.01**
			Day	−0.099	0.260	0.70
			Distance to origo:day	0.013	0.016	0.43
			Heart rate:day	−0.014	0.016	0.38
	Behavioural factors excluded	1725	SEL_cum_	0.000	0.044	0.99
			SEL_sp_	0.013	0.032	0.69
			Day	−0.180	0.730	0.80
			SEL_cum_:day	0.015	0.210	0.50
			SEL_sp_:day	−0.014	0.015	0.36
3) Body acceleration	All covariates		No good models and all covariates were *P* > 0.05			
	Behavioural factors excluded		No good models and all covariates were *P* > 0.05			
4) Distance from origo	All covariates	1273	Swimming depth	−2.200	0.300	**<0.001**
			SEL_cum_	0.130	0.020	**<0.001**
			Heart rate	−0.270	0.120	**0.020**
			Day	4.900	1.800	**<0.01**
			Swimming depth:day	0.420	0.140	**<0.01**
			SEL_cum_:day	−0.059	0.013	**<0.001**
			Heart rate:day	0.069	0.061	0.26
	Behavioural factors excluded	1819	Zero to peak sound pressure level (P_0-pk_)	−0.610	0.110	**<0.001**
			SEL_cum_	0.590	0.110	**<0.001**
			Day	−0.490	−2.000	0.80
			Zero to peak sound pressure level (P_0-pk_):day	0.270	0.060	**<0.001**
			SEL_cum_:day	−0.260	0.060	**<0.001**
SAITHE						
1) Heart rate	Behavioural factors excluded	1209	Zero to peak acceleration level (A_0-pk_)	−0.056	0.130	0.68
			AEL_sp_	0.065	0.170	0.70
			Day	0.470	1.700	0.79
			Zero to peak acceleration level (A_0-pk_):day	−0.003	0.066	0.97
			AEL_sp_:day	0.002	0.077	0.98

## Discussion

Sound exposure from the seismic airgun altered the heart rate of cod during our short-term study. Specifically, fish exhibited reduced heart rate (bradycardia) in response to the PM component of the sound exposure during towed and stationary shooting but not when the boat towed the airgun without shooting. The alteration in heart rate was greatest during the first of the three days of the experiment, suggesting that the cod became habituated to the sound source. However, the weaker correlation between changes in heart rate and the PM component on day 2 may partly have been influenced by increased wave action, which lowered the quality of the PM measurement during the towed shooting that day. For some of the cod, heart rate decreased from 30 to 12 beats per minute during the sound exposure. The decrease could have been to even lower levels, but 10 beats per minute was the lowest level that the heart rate data loggers were able to record. Occasional long intervals, or missing heartbeats, are not unusual in fish. This sort of cardiac inhibition or bradycardia can be elicited by a variety of threatening external stimulus such as exposure to predators, sound, flashing light or erratic movement (Priede, 1983; Karlsen, 1992; Sand and Karlsen, 2000; Cooke *et al.*, 2003). Saithe heart rate was not altered by the sound exposures, but the sample size was low. We acknowledge that heart rate is but one component of cardiac output (i.e., cardiac output is the product of heart rate and stroke volume) and it is possible to change cardiac output by changing only one of its components (Farrell, 1991). There has been much research on the cardiac function of Atlantic cod, and that body of work suggests that heart rate is a less reliable predictor of metabolic rate than stroke volume (or cardiac output; Webber *et al.*, 1998). This does not invalidate our findings but emphasizes their preliminary nature and the fact that one should not automatically assume or infer metabolic costs from heart rate (Thorarensen *et al.*, 1996). We are unaware of research on saithe to inform us on the extent to which heart rate is related to metabolic rate. Nonetheless, heart rate is regarded as a sensitive indicator of stress as it enables researches to characterize the time course of responses to different stressors over several days (Sopinka *et al.*, 2016) and is able to do so in free-swimming fish given its potential to be assessed with biologgers (Cooke *et al.*, 2016).

Both cod and saithe responded to the sound exposures by changing swimming depths and horizontal position in the main sea cage, which likely indicated a flight response, but the reactions differed between the two species and among days. The variable pattern in swimming depths and horizontal position is most likely due to the changing direction of the sound source. The fish behavioural patterns could also be impacted by the fish being held in a conically shaped sea cage with the greatest depth in the centre because fish would tend to be in the centre if they preferred diving to the deepest part of the sea cage. For both cod and saithe, there was a strong correlation between swimming depths and distance to the centre of the sea cage, with fish staying deeper when they stayed close to the centre of the cage. As for the effect on heart rate, the correlation with the day number of exposure suggests that the fish became habituated to the sound exposure.

**Table 5:** Linear mixed effect results for cod temperature-detrended heart rate’s relationship to sound measurements interacting (:) with day for 20 HRT (heart rate) tagged fish, excluding the behavioural data from the acoustic tags. Separate models were tested for each sound measurement

Response variable	Covariate name	Delta AICc_null_	Coefficient	SE	*P*
Heart rate	Zero to peak sound pressure level (A_0-pk_)	9846	−0.240	0.030	**<0.001**
	Zero to peak sound pressure level (A_0-pk_):day		0.077	0.014	**<0.001**
	Zero to peak sound pressure level (P_0-pk_)	9852	−0.210	0.025	**<0.001**
	Zero to peak sound pressure level (P_0-pk_):day		0.068	0.010	**<0.001**
	AEL_cum_	9875	−0.180	0.032	**<0.001**
	AEL_cum_:day		0.061	0.015	**<0.001**
	AEL_sp_	9879	−0.160	0.032	**<0.001**
	AEL_sp_:day		0.045	0.015	**<0.01**
	Single pulse sound pressure level (SEL_sp_)	9895	−0.140	0.024	**<0.001**
	Single pulse sound pressure level (SEL_sp)_:day		0.040	0.100	**<0.001**
	Cumulative pulse sound pressure level (SEL_sp_)	9859	−0.220	0.027	**<0.001**
	Cumulative pulse sound pressure level (SEL_sp_):day		0.073	0.011	**<0.001**

When the cod dived to greater depths, the heart rate increased, which could be due to several factors including warmer water at depth. Heart rate may change with changing body temperature; however, there is a delay in warming and cooling of the fish body when it moves to water layers with a different temperature (Spigarelli *et al.*, 1977). This suggests that the increased heart rate during dives was directly connected to the diving activity or the sound exposure and not to the temperature change. The sound exposure might have caused both diving to deeper depths and an increased heart rate. Other studies have shown that fish may move to deeper depths during stress (Papandroulakis *et al.*, 2014; Neo *et al.*, 2018). No significant diving behaviour after sound exposures was observed in the video data. However, the maximum 4 m that fish could have descended in this smaller pen precluded them access to the deeper and darker sections of the water column used by the fish in the large cage.

Previous studies of free-swimming fish have shown that sound exposure can induce a startle response (Chapman and Hawkins, 1969; Wardle *et al.*, 2001; Hawkins *et al.*, 2014). The body acceleration data gathered in this experiment provided no indication of a startle response by the fish, a finding that was supported by the video observations. It may be that no such effect was observed due to the simulated ramp-up protocol employed, with sound levels gradually increasing as the boat towed the airgun closer. However, if so, an increase in body acceleration could be expected during the stationary shooting with no simulated ramp up, but this was not the case. It is important to highlight that the strongest response observed by Wardle *et al.* (2001) was associated with combined sound and visual stimuli from the airgun. In their experiment the close proximity of the seismic airgun meant that fish could see the bubbles and clouds of sand created during shooting. All observed fish showed a C-start response, an escape reflex initiated by a rapid bending of the body into a ‘C’ shape (Domenici and Blake, 1997), followed by a directional change. When the airgun was located at least 90 m away and not visible, fish exhibited a C-start side skip reflex but did not change swimming direction (Wardle *et al.*, 2001). In the current study, there was no possibility of visual cues. Alternatively, the noisy baseline environment at the site may have to some degree conditioned the fish to loud noises, reducing the propensity for startle responses. We also acknowledge that C-start occurs over fractions of a second and therefore may not have been recorded in our footage if it was not followed by a significant change in swimming direction.

Like the changes in position during the sound exposures observed using acoustic telemetry, the video footage showed that the saithe in the smaller net pen became more dispersed and less aligned with the principal flow direction when exposed to sound from the airgun, particularly during stationary shooting. Under normal conditions, saithe are one of the strongest facultative schoolers among gadoid fishes, tending to form schools where neighbours align and swim at a similar depth (Partridge *et al.*, 1980). The breakdown in schooling is strong evidence for a response to the stimulus even in the absence of visual cues associated with the airgun firing or the boat. Schooling is a normal anti-predator behaviour, and a breakdown in schooling like those observed here could imply a greater predation risk caused by the sound exposure. However, this will depend on many factors such as fish size and the presence of predators and would be difficult to demonstrate and quantify in a natural situation.

The levels of sound pressure and particle acceleration, measured at the edge of the sea cage during towed and stationary airgun shooting, were above earlier reported levels (P_0-pk_ 140–161 dB re 1 μPa; A_0-pk_ 57–76 dB re 1 μm/s^2^) that induced a behavioural reaction in cod exposed to playbacks of pile driving sounds (Mueller-Blenkle *et al.*, 2010). Habituation effects like those seen over time in this study were also noted by Mueller-Blenkle *et al.* (2010).

In conclusion, we revealed that airgun shooting was associated with a period of bradycardia (lowered heart rate) in cod, consistent with the onset of a stress response, but this was not followed by a period of tachycardia or arrhythmia, which is typically expected in the event of a prolonged response. Therefore, sound induced a short-term physiological stress response in cod, but they quickly recovered and habituated after repeat exposure. Consequently, the sound exposures induced in this study may not be associated with long-term alterations in fish physiology. For saithe, heart rate was reasonably stable when exposed to sound but the sample size was low. We also revealed that swimming depth and change in position, but not body acceleration, were influenced by the sound exposures in both cod and saithe and was most likely an indication of a flight response.

Our study suffered from several inherent limitations, most notably relatively low sample sizes. Stormy weather conditions on the second day of the study may have increased the variability of responses observed. Clearly, additional research is needed that includes a larger number of fish spanning a greater range of environmental conditions. The short-term study should be followed up by an experiment including several groups of independent exposure and a control group. Although the fish studied here were free-swimming, they were still confined to a cage. Including observations on fish not confined to a cage would be advantageous in addition to controlled laboratory or mesocosm experiments, since even a large sea cage could inhibit a potential flight response. Nonetheless, it is important to recognize that this type of research is quite difficult given the reality of attempting experimental biology in the open ocean and even the preliminary research that we presented here represents a massive endeavour involving partnerships with industry and scientists. Additional research and creativity is needed to fully understand the effects of different anthropogenic noise sources on wild fish.

## Author contributions

Davidsen, Dong, Piper, Thorstad, Whoriskey, Cooke, Sjursen, Rønning and Hawkins conceived the ideas and designed the methodology; Davidsen, Linné, Andersson, Piper, Sjursen, Rønning and Netland collected the data; Davidsen, Dong, Linné, Andersson, Piper, Prystay and Hvam analysed data; and Davidsen led the writing of the manuscript. All authors contributed to the drafts and approved publication.

## Supplementary Material

Supp_coz020
