# Supplementary material for: Effects of sound exposure from a seismic airgun on heart rate, acceleration and depth use in free-swimming Atlantic cod and saithe
Source: Conserv Physiol. 2019 May 16;7(1):coz020. doi: 10.1093/conphys/coz020 (PMC6521782; doi:10.1093/conphys/coz020)
Supplement: Supp_coz020 [file supp_coz020.pdf]

Table S1: Overview of sound exposures in Vinjefjorden 21-23 November 2017. Type of exposure: *Transect* means that the airgun was towed 11 m behind the boat at 4 m depth at a speed of 8.1 8.5 km h^{-1}, with a shooting sequence of 1 shoot per 10 s. *Towing of airgun, no shooting* means that the airgun was towed from the last position of the active shooting transect to the mooring location. Boat speed and airgun depth were similar to those used during the *transects*. *Stationary* means that the boat was moored during the shooting sequence. Time and bar pressure of first and last shooting of the sound exposures are also given.

For Review Only

**Date**	**Exposure number**	**Type of sound exposure**	**First shoot (UTC and bar pressure of airgun)**	**Last shoot (UTC and bar pressure of airgun)**	**Times of towing of airgun, no shooting (UTC)**
**21.11.2017**	1	Transect	12:07 = 138 bar	13:13 = 138 bar	
**21.11.2017**	2	Towing of airgun, no shooting	NA	NA	14:07-14:35
**21.11.2017**	3	Stationary	14:37 = 125 bar	14:43 = 100 bar	
**22.11.2017**	4	Transect	09:29 = 135 bar	10:29 = 135 bar	
**22.11.2017**	5	Towing of airgun, no shooting	NA	NA	10:33-10:40
**22.11.2017**	6	Stationary	13:17 = 130 bar	13:27 = 125 bar	
**23.11.2017**	7	Transect	11:24 = 138 bar	12:26 = 138 bar	
**23.11.2017**	8	Towing of airgun, no shooting	NA	NA	12:28-12:40
**23.11.2017**	9	Stationary	13:53 = 138 bar	14:03 = 138 bar	

Table S2. Definitions of equations from the ISO 18405 standard that were used for the different calculations related to the sound pressure and particle acceleration analysis.

ISO 18405 Chapter	Definition	Abbreviation	Unit
**3.2.1.9**	Sound Exposure Spectral Density	Sound ESD	dB re 1 μPa^2·s/Hz
**3.2.1.5**	Sound Exposure Level	$SEL_{sp/cum}$	dB re 1 μPa^2·s
**3.2.2.1**	Zero to Peak Sound Pressure Level	$P_{0\text{-}pk}$	dB re 1 μPa
**-**	Acceleration Exposure Spectral Density level	Acceleration ESD	dB re 1 (μm/s^2)^2·s/Hz
**-**	Acceleration Exposure Level	$AEL_{sp/cum}$	dB re 1 (μm/s^2)^2·s
**-**	Zero to Peak Acceleration Level	$A_{0\text{-}pk}$	dB re 1 μm/s^2

For Review Only

Table S3. Daily resting heart rate (beats per minute) for each individual. Resting heart rate was calculated as the mean of the lowest 10% heart rate values recorded each day.

Fish ID	2017-11-20	2017-11-21	2017-11-22	2017-11-23	2017-11-24	Mean
**1**	15	14.54167	14.15278	15.30556	15.44444	**14.83333**
**2**	27.06944	25.29167	22.15278	22.51389	24.68056	**23.29398**
**3**	16.40278	16.72222	15.27778	18.34722	16.36111	**16.32407**
**4**	16.52778	15.91667	14.20833	16.38889	16.47222	**15.55556**
**5**	21.05556	21.95833	19.19444	19.79167	19.47222	**19.2662**
**6**	19.13889	19.09722	18.30556	16.73611	17.51389	**17.71991**
**7**	22.84722	22.88889	21.01389	22.68056	22.31944	**21.90972**
**8**	24.25	25.98611	28.69444	36.44444	39.88889	**25.26852**
**9**	17.51389	16.66667	17.04167	17.31944	16.77778	**17.21296**
**10**	20.25	20.22222	19.79167	21.30556	20.59722	**20.51157**
**11**	NA	NA	NA	NA	NA	**NA**
**12**	14.94444	14.80556	13.19444	14.22222	14.22222	**14.18981**
**13**	34.11111	30.73611	29.93056	30.375	35.66667	**31.07176**
**14**	27.98611	28.93056	27.73611	28.54167	29.70833	**28.36574**
**15**	26.79167	30.06944	27.54167	27.875	32.63889	**27.72685**
**16**	NA	NA	NA	NA	NA	**NA**
**17**	NA	NA	NA	NA	NA	**NA**
**18**	NA	NA	NA	NA	NA	**NA**
**19**	NA	NA	NA	NA	NA	**NA**
**20**	NA	NA	NA	NA	NA	**NA**
**21**	NA	NA	NA	NA	NA	**NA**
**22**	NA	NA	NA	NA	NA	**NA**
**23**	15.66667	18.18056	17.05556	18.25	18.56944	**16.31481**
**24**	NA	NA	NA	NA	NA	**NA**
**25**	19.06944	20.97222	18.95833	18.55556	20.93056	**18.12731**
**26**	NA	NA	NA	NA	NA	**NA**
**27**	22.88889	20.15278	19.20833	18.77778	18.83333	**19.19192**
**28**	26.44444	22.25	20.45833	19.36364	NA	**20.50761**
**29**	26.77778	22.66667	18.84722	18	20.47222	**19.09091**
**30**	34.11111	22.86111	23.26389	25.02778	24.59722	**23.78114**

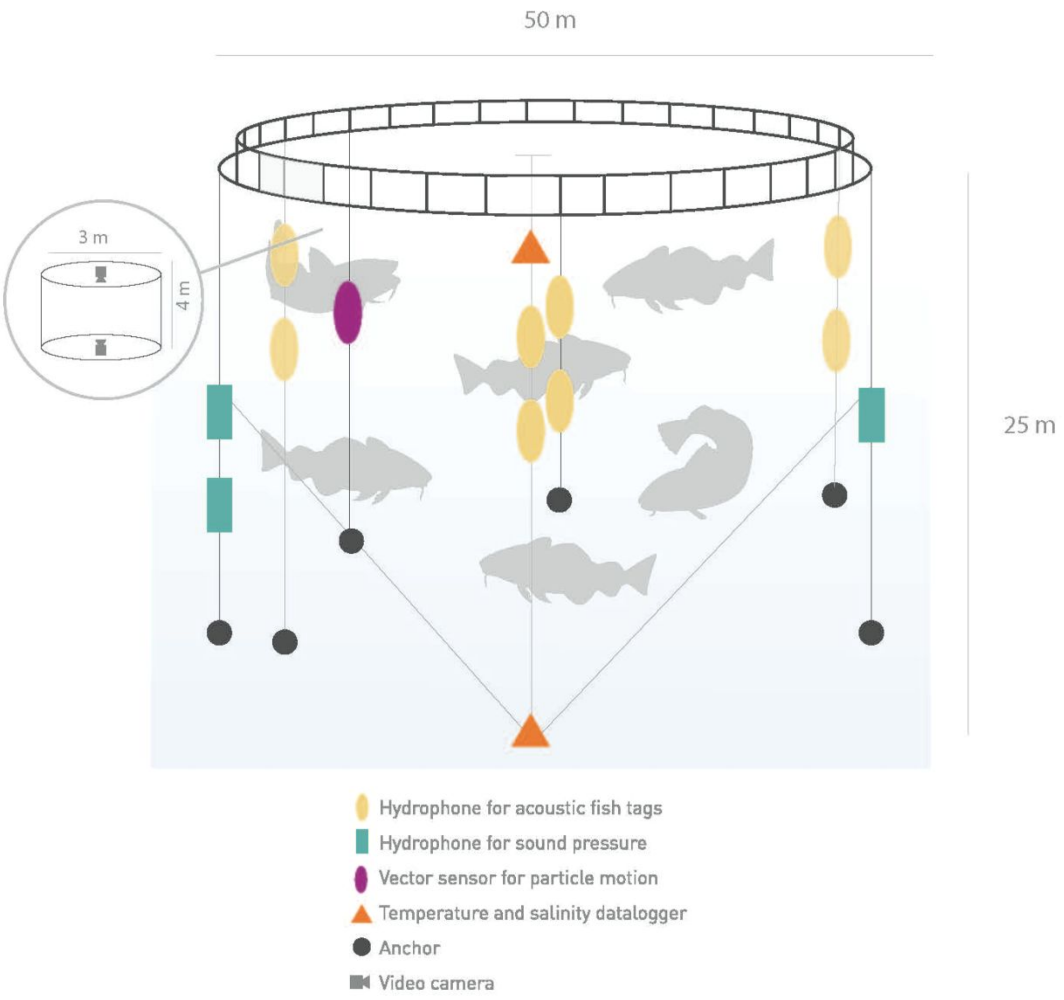

Figure S1: Location of various sensors in the sea cage during the sound exposure experiment. The boat towing the airgun arrived from what is shown as the left side of the pen in the figure, passed around the sea cage, first on the front side and then on the back side of the pen, and then exited on the left side. The small net pen indicated by the insert on the left side was equipped with a video monitoring system and placed on the western (left) side of the large sea cage.

756

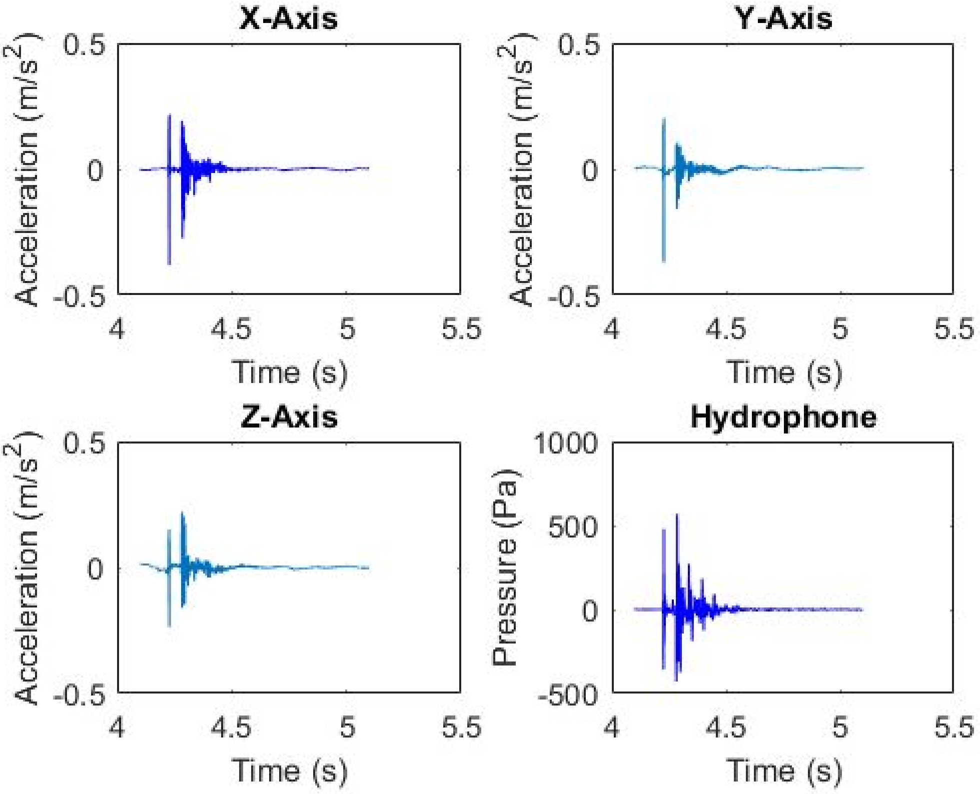

757 Figure S2. Time series of an airgun pulse when the airgun was stationary 200 m away, showing the

758 acceleration at the sea cage for the three axes and the hydrophone output.

For Review Only

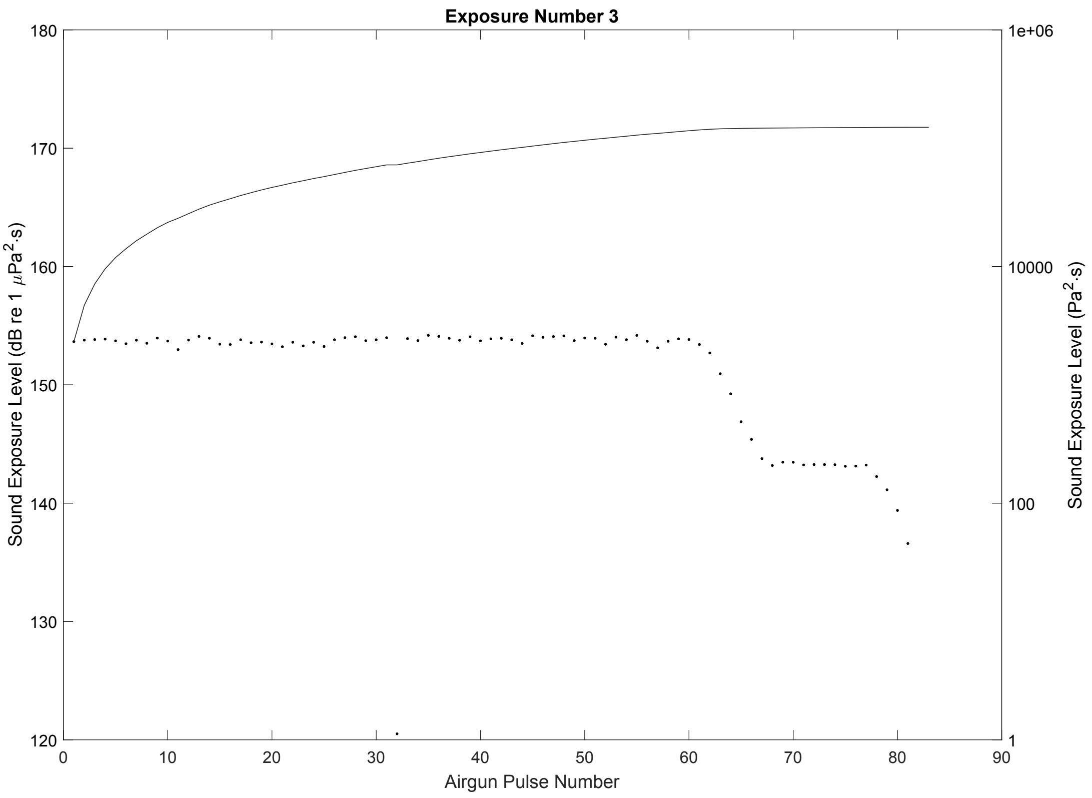

Figure S3. Sound exposure level (single pulse and cumulative) when the airgun was stationary 200 away from the sea cage during exposure 3 on day 1. The dots represent the SEL_{sp} and the curve the SEL_{cum}.

iew Only

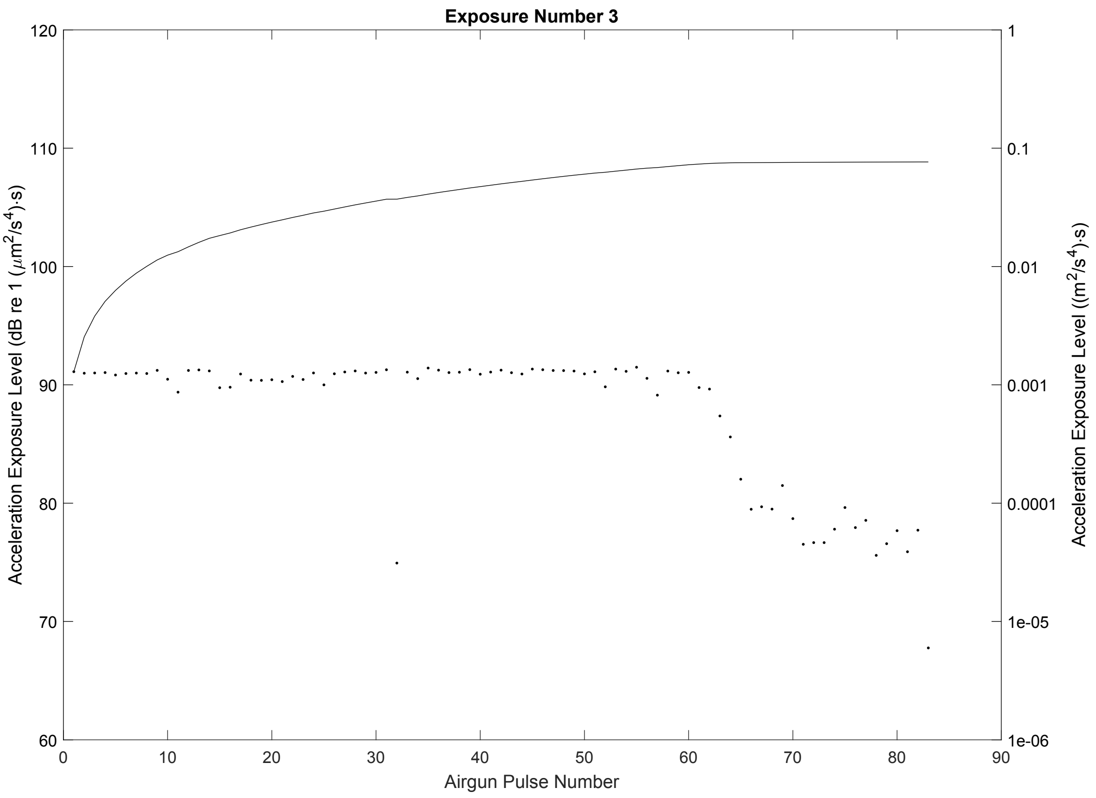

Figure S4. Acceleration exposure level (single pulse and cumulative) when the airgun was stationary, exposure number 3 (day 1), 200 away from the sea cage. The dots represent the AEL_{sp} and the curve the AEL_{cum}.

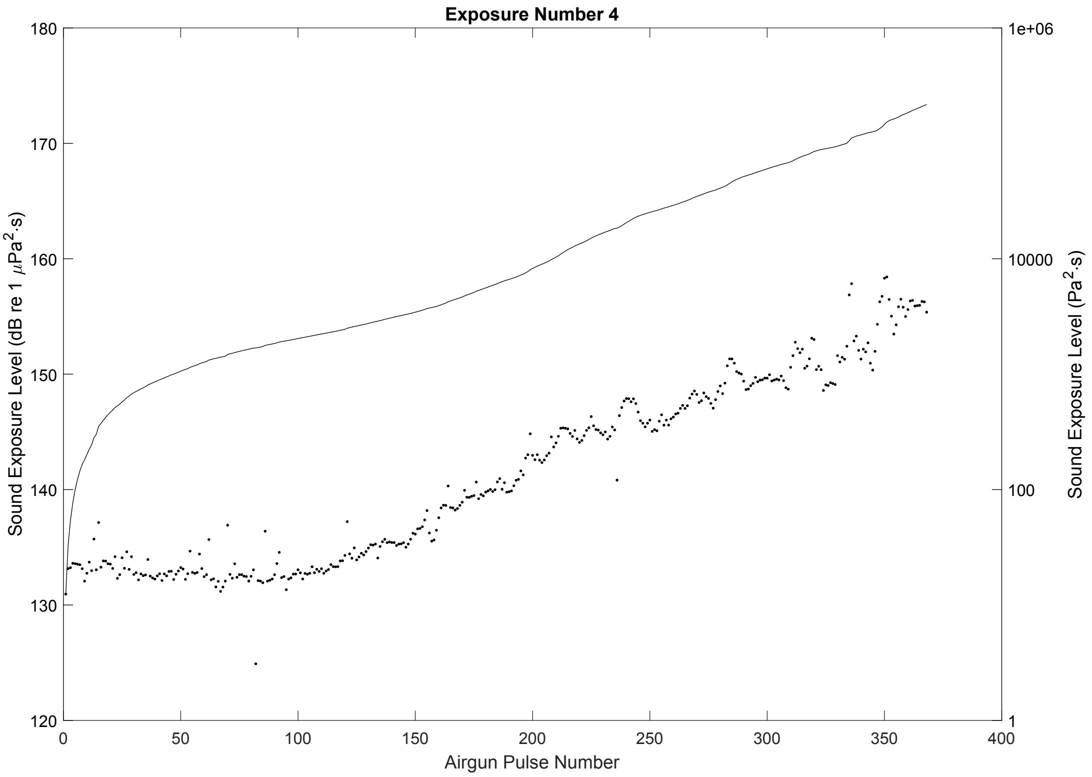

Figure S5. Sound exposure level (single pulse and cumulative) when the airgun was moving 100-6700 m away from the sea cage during exposure 4 on day 2. The dots represent the SEL_{sp} and the curve the SEL_{cum}.

view Only

777

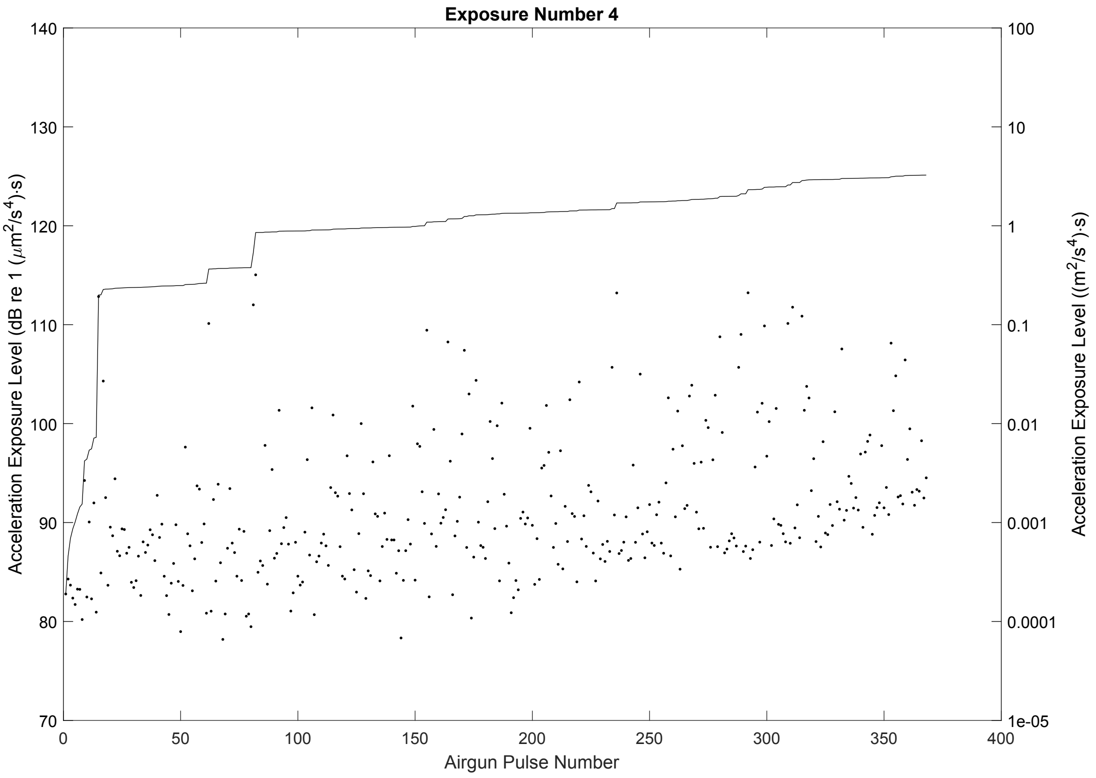

778 Figure S6. Acceleration exposure level (single pulse and cumulative) when the airgun was moving
779 100-6700 m away from the sea cage during exposure number 4 on day 2. The dots represent the AEL_{sp}
780 and the curve the AEL_{cum}.

view Only

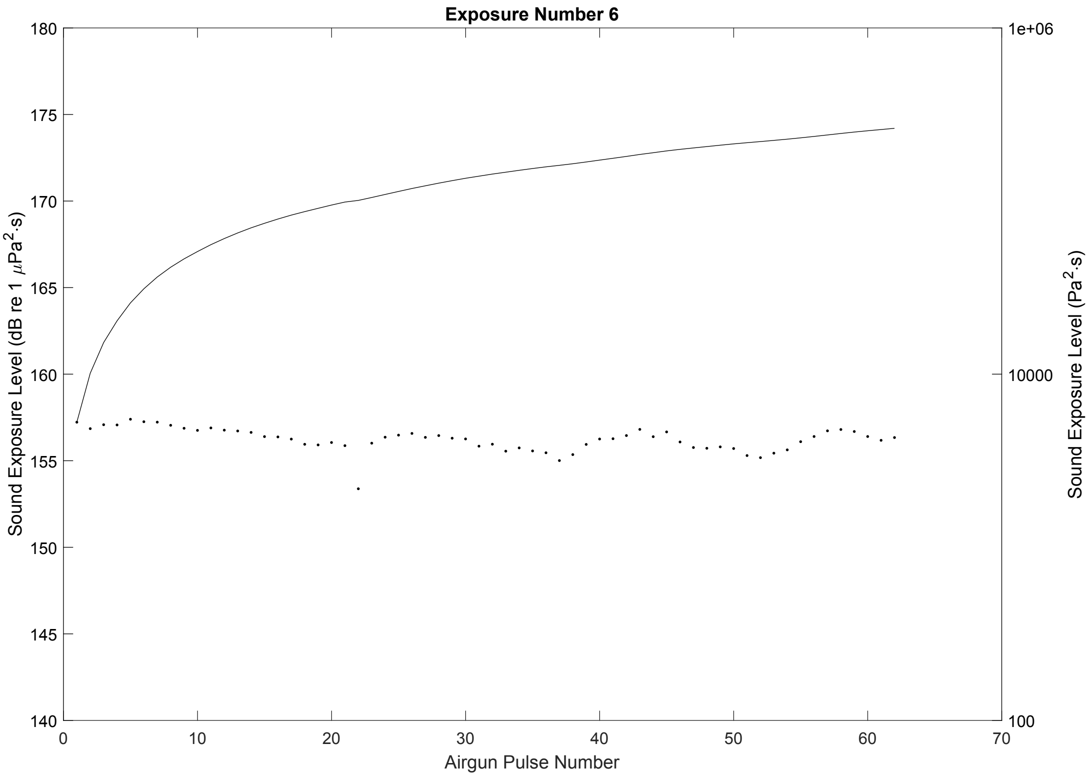

Figure S7. Sound Exposure Level (single pulse and cumulative) when the airgun was stationary 200 m away from the sea cage during exposure 6 on day 2. The dots represent the SEL_{sp} and the curve the SEL_{cum}.

view Only

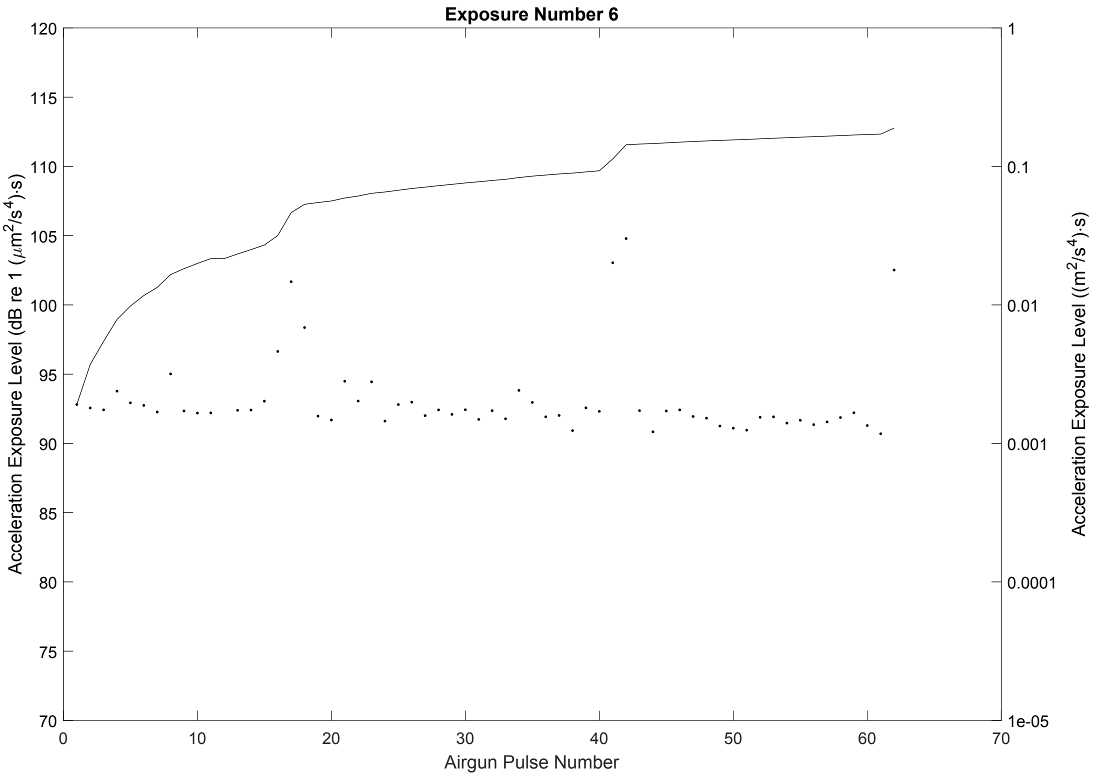

Figure S8. Acceleration Exposure Level (single pulse and cumulative) when the airgun was stationary 200 m away from the sea cage during exposure 6 on day 2. The dots represent the AEL_{sp} and the curve the AEL_{cum}.

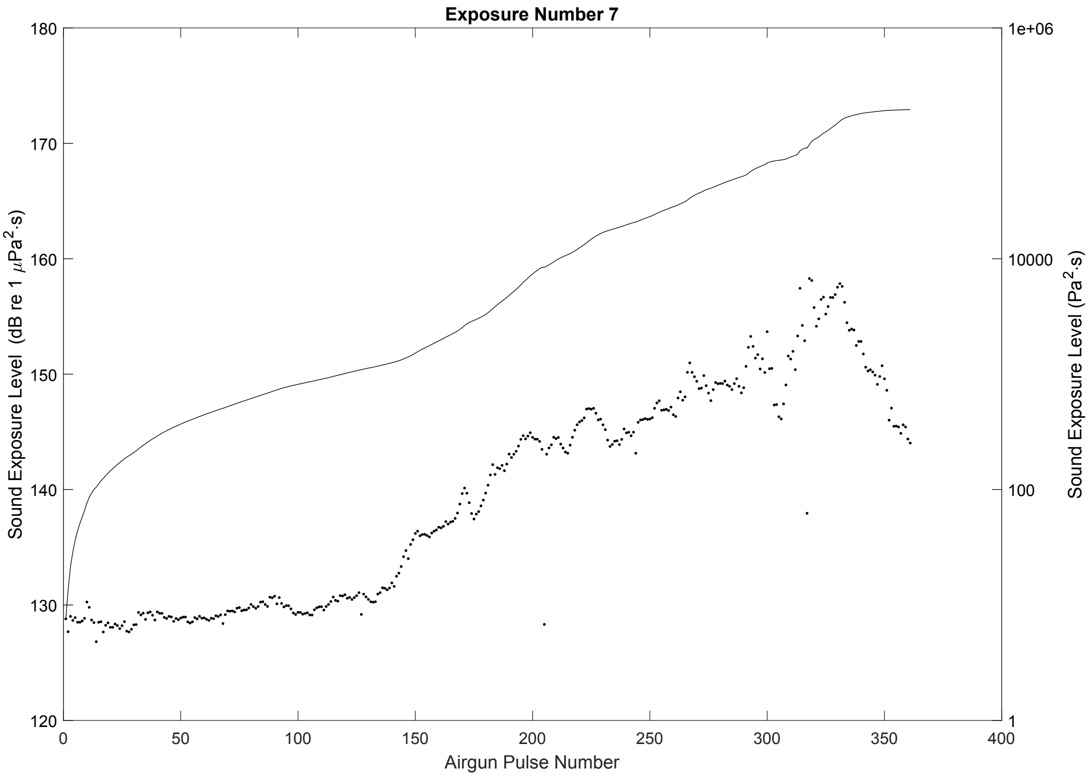

Figure S9. Sound Exposure Level (single pulse and cumulative) when the airgun was moving 100-6700 m away from the sea cage during exposure 7 on day 3. The dots represent the SEL_{sp} and the curve the SEL_{cum}.

view Only

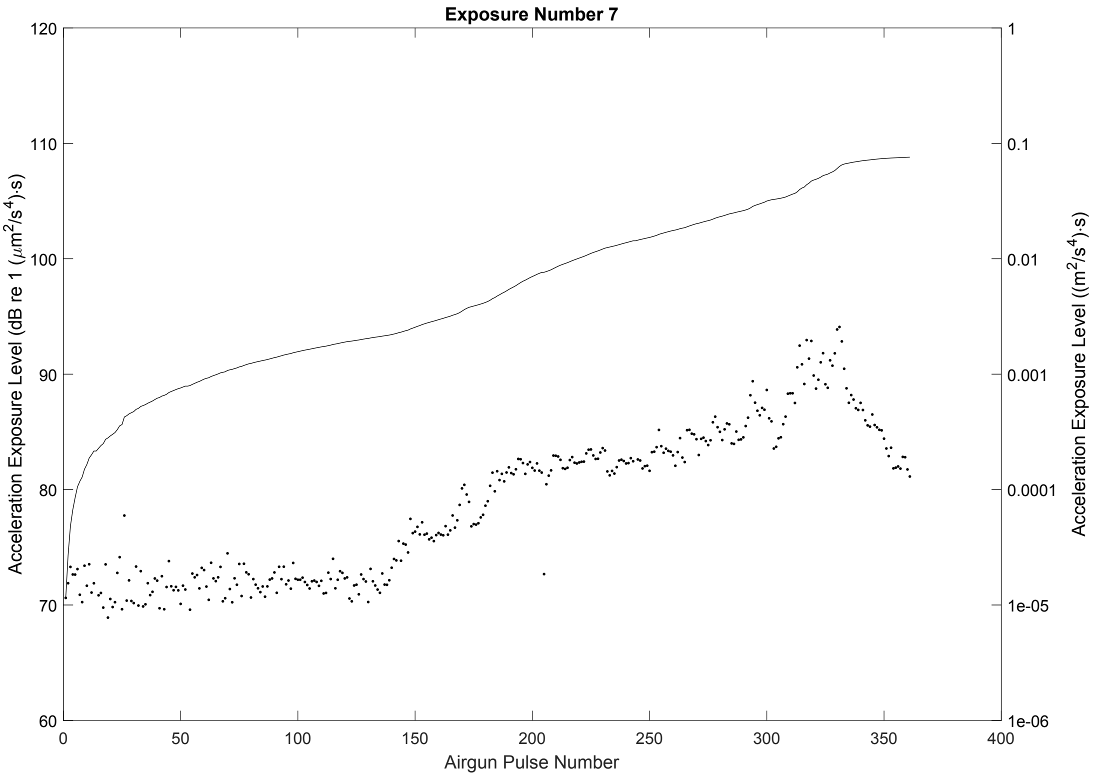

Figure S10. Acceleration Exposure Level (single pulse and cumulative) when the airgun was moving 100-6700 m away from the sea cage during exposure 7 on day 3. The dots represent the AEL_{sp} and the curve the AEL_{cum}.

view Only

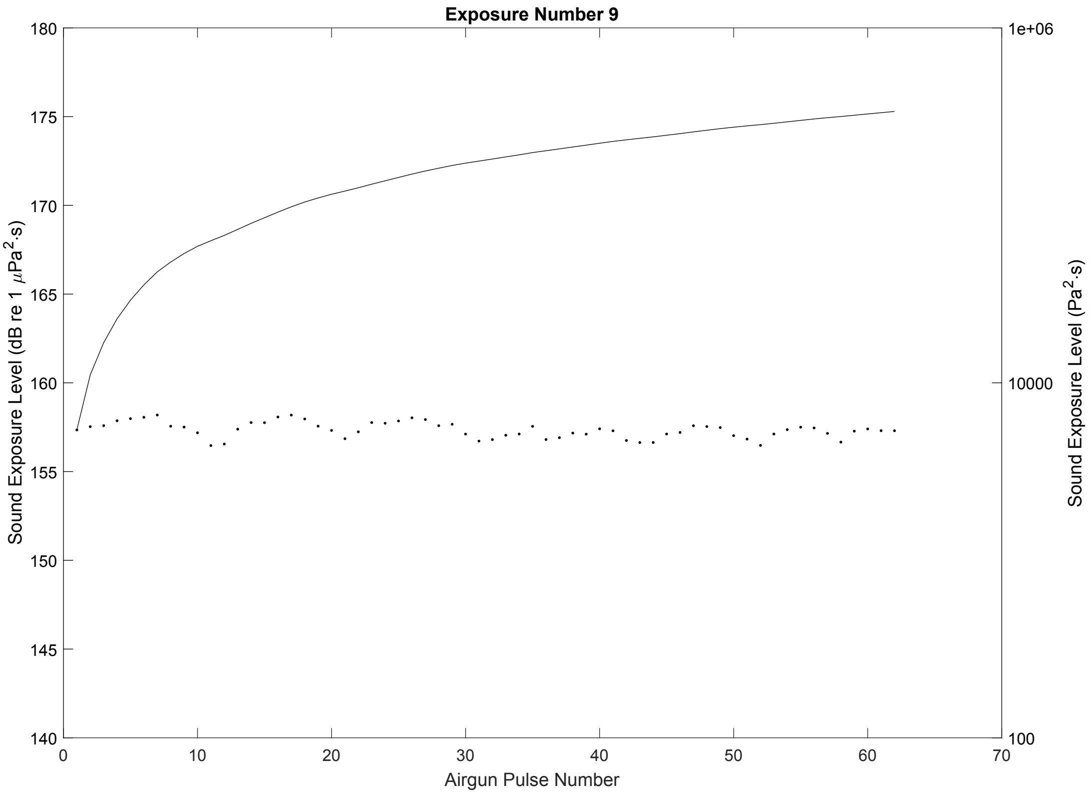

Figure S11. Sound Exposure Level (single pulse and cumulative) when the airgun was stationary 200 m away from the sea cage during exposure 9 on day 3. The dots represent the SEL_{sp} and the curve the SEL_{cum}.

iew Only

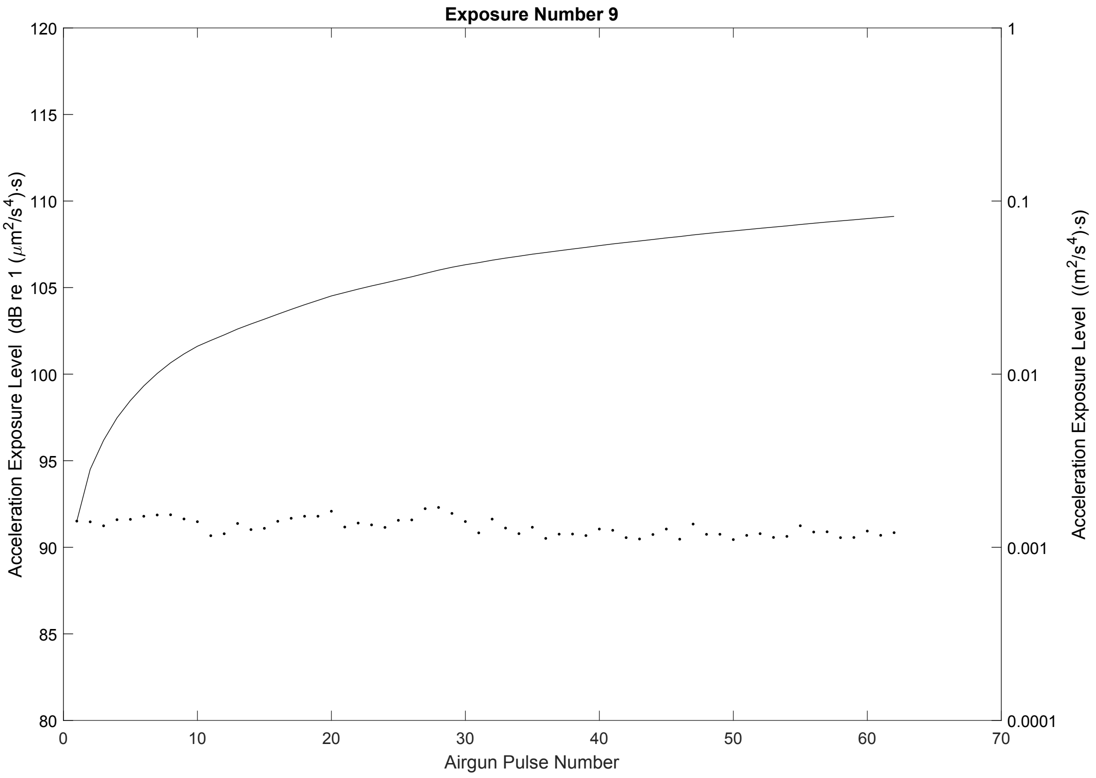

Figure S12. Acceleration Exposure Level (single pulse and cumulative) when the airgun was stationary 200 m away from the sea cage during exposure 9 on day 3. The dots represent the AEL_{sp} and the curve the AEL_{cum}.

view Only

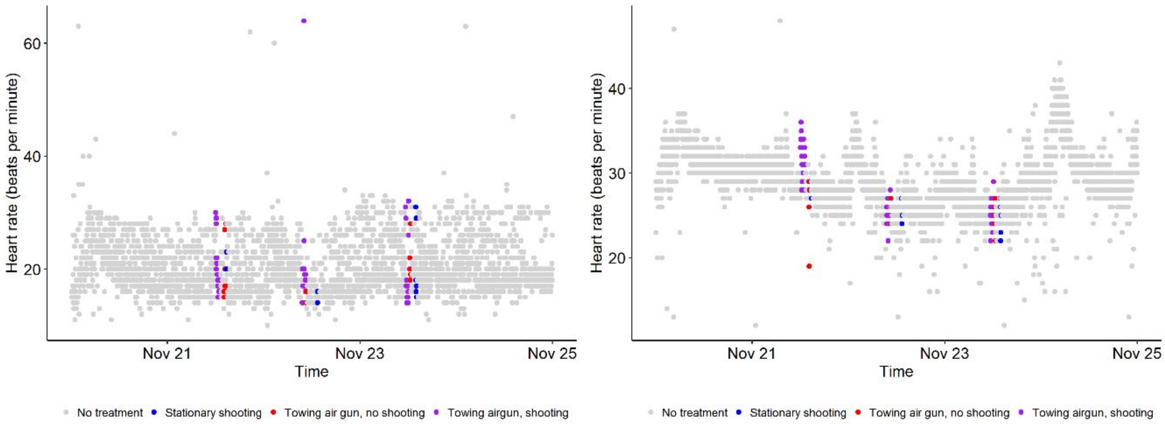
Heart rate (beats per minute)
60
40
20
Nov 21
Nov 23
Nov 25
Time
No treatment
Stationary shooting
Towing air gun, no shooting
Towing airgun, shooting
Heart rate (beats per minute)
40
30
20
Nov 21
Nov 23
Nov 25
Time
No treatment
Stationary shooting
Towing air gun, no shooting
Towing airgun, shooting

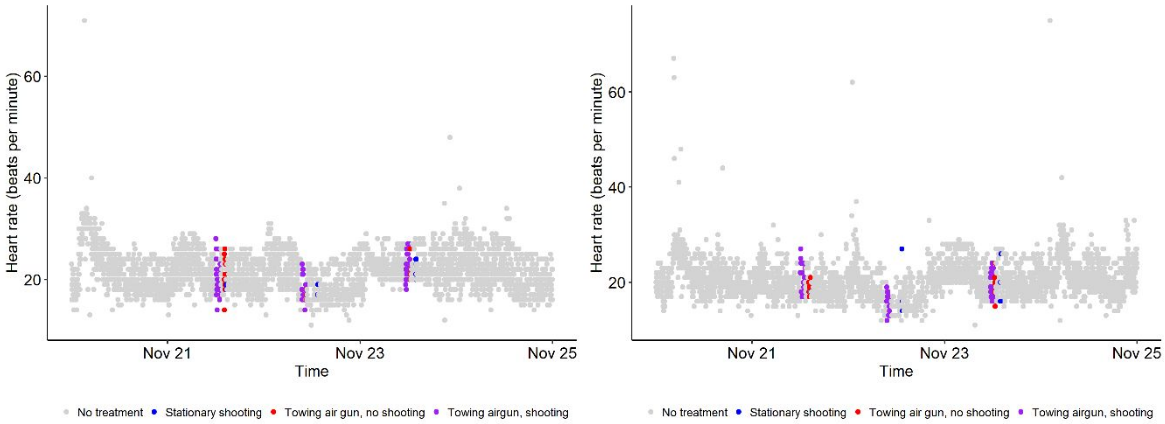
Heart rate (beats per minute)
60
40
20
Nov 21
Nov 23
Nov 25
Time
No treatment
Stationary shooting
Towing air gun, no shooting
Towing airgun, shooting
Heart rate (beats per minute)
60
40
20
Nov 21
Nov 23
Nov 25
Time
No treatment
Stationary shooting
Towing air gun, no shooting
Towing airgun, shooting

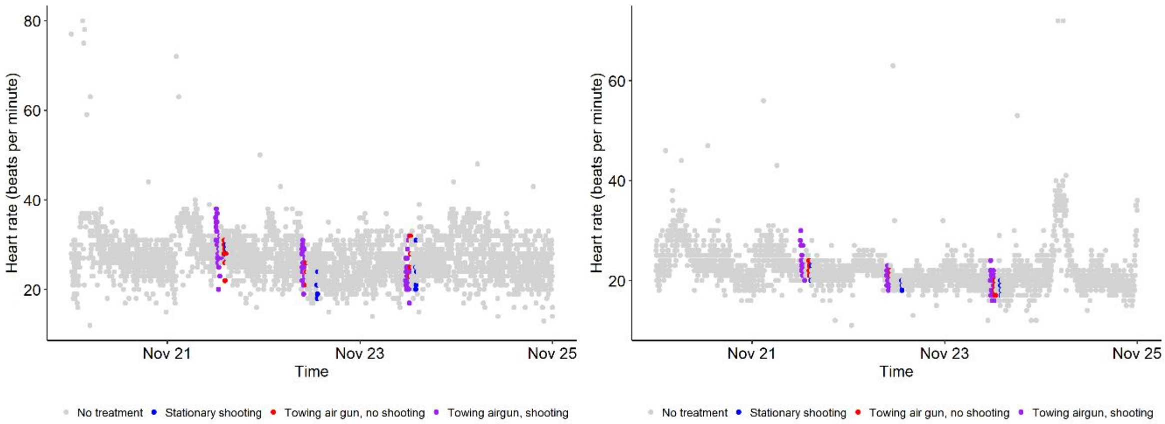
Heart rate (beats per minute)
80
60
40
20
Nov 21
Nov 23
Nov 25
Time
No treatment
Stationary shooting
Towing air gun, no shooting
Towing airgun, shooting
Heart rate (beats per minute)
60
40
20
Nov 21
Nov 23
Nov 25
Time
No treatment
Stationary shooting
Towing air gun, no shooting
Towing airgun, shooting

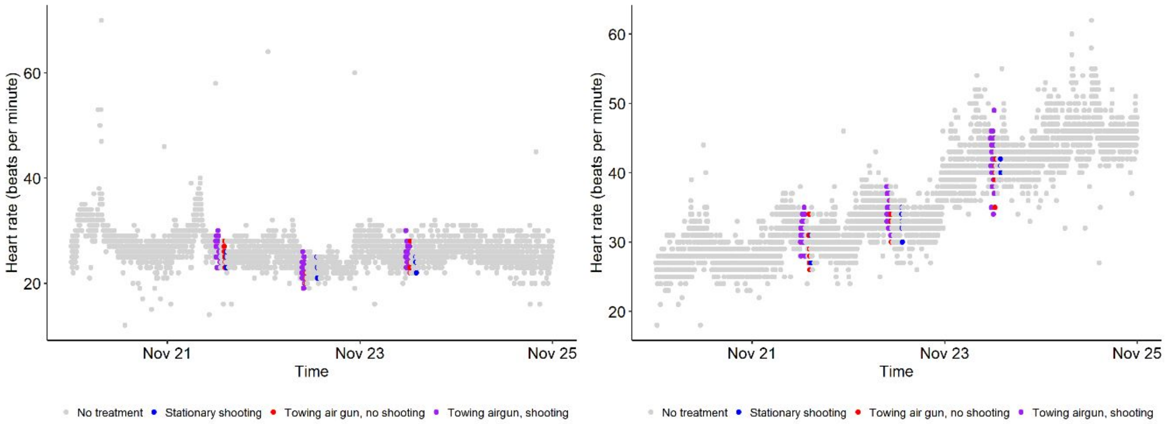
Heart rate (beats per minute)
60
40
20
Nov 21
Nov 23
Nov 25
Time
No treatment
Stationary shooting
Towing air gun, no shooting
Towing airgun, shooting
Heart rate (beats per minute)
60
50
40
30
20
Nov 21
Nov 23
Nov 25
Time
No treatment
Stationary shooting
Towing air gun, no shooting
Towing airgun, shooting

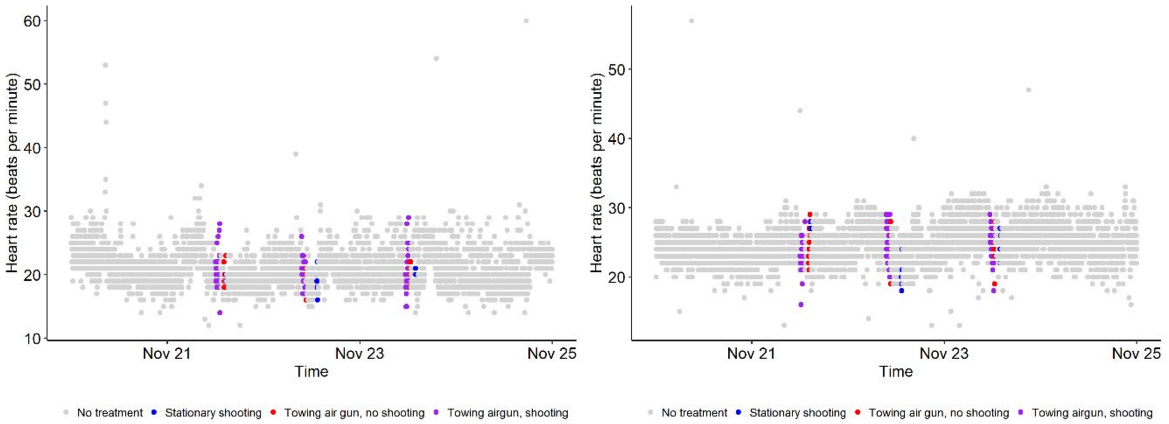

812

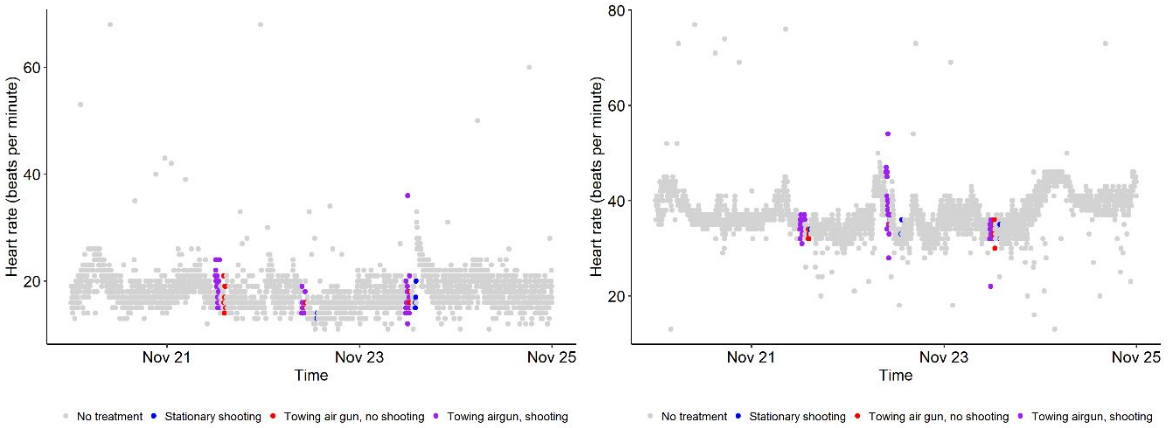

813

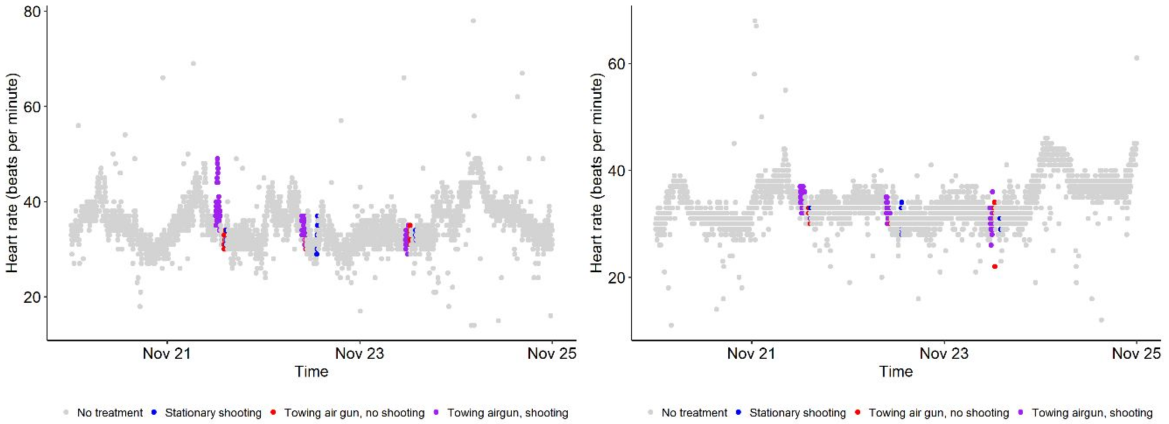

814

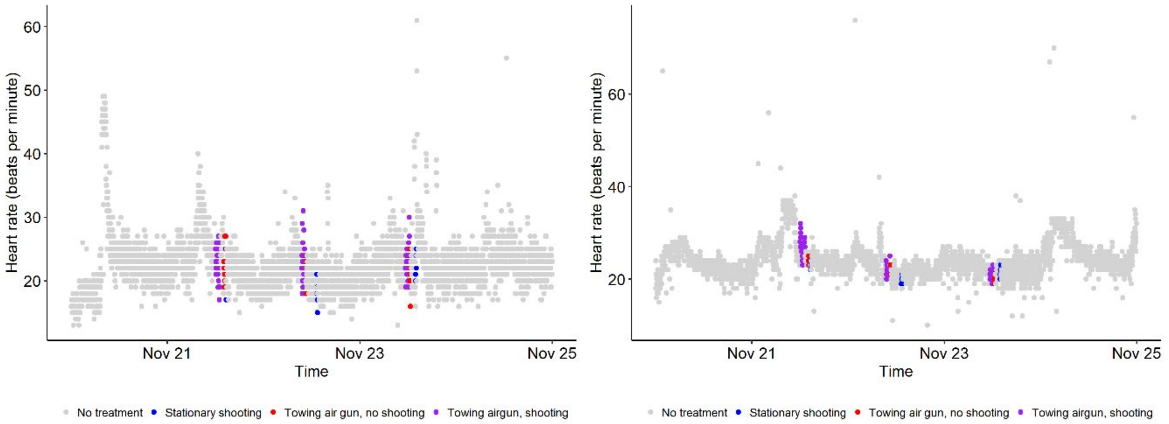

815

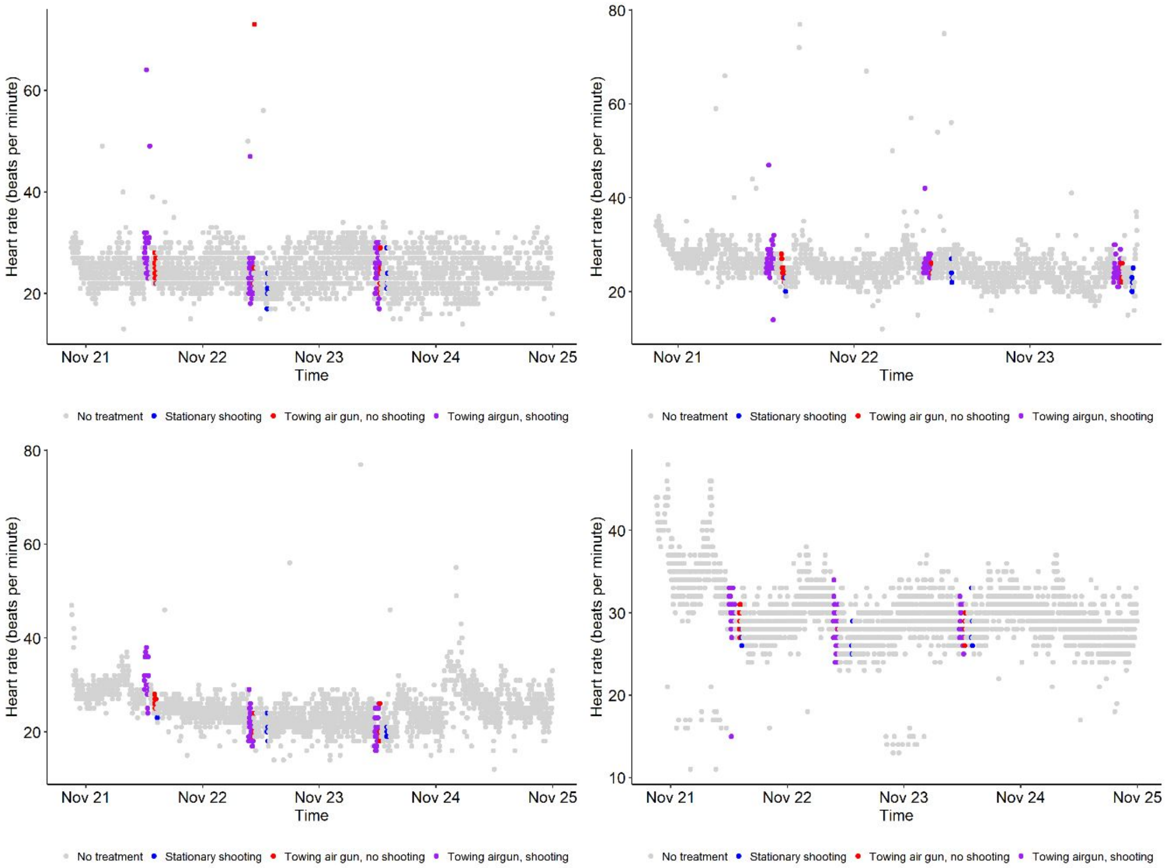

Figure S13. Heart rate traces of indivudal fish during the experimental period. Heart rate traces start November 20, 2017 a midnight and end November 25, 2017 a midnight.
